# CicerTransDB 1.0: a resource for expression and functional study of chickpea transcription factors

**DOI:** 10.1186/s12870-016-0860-y

**Published:** 2016-07-29

**Authors:** Saurabh Gayali, Shankar Acharya, Nilesh Vikram Lande, Aarti Pandey, Subhra Chakraborty, Niranjan Chakraborty

**Affiliations:** National Institute of Plant Genome Research, Jawaharlal Nehru University Campus, Aruna Asaf Ali Marg, New Delhi, 110067 India

**Keywords:** Chickpea, Database, Food legume, Genome-wide, Transcription factor

## Abstract

**Background:**

Transcription factor (TF) databases are major resource for systematic studies of TFs in specific species as well as related family members. Even though there are several publicly available multi-species databases, the information on the amount and diversity of TFs within individual species is fragmented, especially for newly sequenced genomes of non-model species of agricultural significance.

**Description:**

We constructed CicerTransDB (Cicer Transcription Factor Database), the first database of its kind, which would provide a centralized putatively complete list of TFs in a food legume, chickpea. CicerTransDB, available at www.cicertransdb.esy.es, is based on chickpea (*Cicer arietinum* L.) annotation v 1.0. The database is an outcome of genome-wide domain study and manual classification of TF families. This database not only provides information of the gene, but also gene ontology, domain and motif architecture.

**Conclusion:**

CicerTransDB v 1.0 comprises information of 1124 genes of chickpea and enables the user to not only search, browse and download sequences but also retrieve sequence features. CicerTransDB also provides several single click interfaces, transconnecting to various other databases to ease further analysis. Several webAPI(s) integrated in the database allow end-users direct access of data. A critical comparison of CicerTransDB with PlantTFDB (Plant Transcription Factor Database) revealed 68 novel TFs in the chickpea genome, hitherto unexplored.

Database URL: http://www.cicertransdb.esy.es

**Electronic supplementary material:**

The online version of this article (doi:10.1186/s12870-016-0860-y) contains supplementary material, which is available to authorized users.

## Background

Chickpea is one of the most important pulse crops with a diverse array of potential nutritional and health benefits. It is the world’s second most widely grown legume after soybean. Currently, chickpea is grown in over fifty countries covering ~12771 million hectares [Faostat, 2013] and has become a leading crop in many nations across the globe [1]. Chickpea has significant amounts of all the essential amino acids that cannot be synthesized by the human body [2]. Due to its high value in the agro-industries, chickpea has received much research attention, particularly in the areas of improvement in agronomic performances and as a model for basic biological studies. Chickpea is presumed to be of special importance to food security for the developing world wherein, due to its biological nitrogen fixation capability, it is a primary source of human dietary protein [1].

The genome of chickpea was sequenced and assembled by whole-genome shotgun sequencing [1]. Despite progress in the functional annotation of the genes, analysis platforms for accessing detailed information from the draft sequence are limited. This level of annotation also holds true for transcription factors (TFs). The regulation of gene expression by TFs is crucial to growth and development as well as the physiology of plants. It is now known that TFs weave a complex inter-regulatory network within the cell that influences almost all metabolic processes. TFs recognize specific DNA sequences that are the key components involved in gene regulatory networks, and are therefore of particular interest for functional characterization.

*Arabidopsis* is the first plant to be completely sequenced, which provides the foundation for identifying a wide range of plant-specific gene functions. Transcription factors of *Arabidopsis* have been well studied [3–5], which made it possible to identify and study TFs of other sequenced plant species by homology searching and comparative analysis. The available databases provide: (1) a uniform platform to review plant TF families across species; (2) descriptions of each TF family and links to the appropriate literature; and (3) cross-references between the databases by means of orthologous relationships.

The transcription process in eukaryotes is mediated by the general transcription factors (GTFs), the protein factors involved in messenger RNA synthesis [6, 7], which are conserved across species. The cataloguing of plant-specific TFs was initiated with the release of TRANSFAC (Transcription Factor Database) database extensively represented by cis-acting elements and trans-acting factors of Arabidopsis [8]. Currently available plant-specific GTF databases can be exemplified by PlantTFDB (Plant Transcription Factor Database) [9] and DBD (DNA-binding Domain) [10], comprising information about TFs from multiple plant species. However, such databases poorly represent the newly sequenced genomes, for example, the TFs of chickpea. Traditional method for prediction of TFs, specifically organism-specific TFs, uses blast homology or pattern-specific (hmm) search on the complete genome. The former method is greatly biased to the seed database used for searching against the genome and therefore, very often lacks discovery of new TFs with novel sequences. The latter method is relatively slow, as the initial seed for domain-specific blast (PSI-BLAST or hmmer) needs seeds of their own, making database generation and maintenance a cumbersome process.

In this study, we used a quick and accurate method of whole genome cataloguing for generation of chickpea TF database. CicerTransDB is a database of TFs of chickpea discovered by the process of cataloguing domains of chickpea gene-products using domain-specific seeds from pfam. It harbours 1124 chickpea TFs grouped into 47 separate families. The database expands to features like motifs, domains, homologues in PlantTFDB and TAIR, gene ontology, among others which in turn gives the user a better interface for quick research in comparison to general TF databases. The database also takes care of need to analyse information in other databases. Additionally, various databases can be directly queried, for example, InterProScan, PlantCare and many more. Direct blast submission to NCBI and ENA databases has also been facilitated through a single click interface. These tools along with sequence information make CicerTransDB a comprehensive platform to visualize and cross-search chickpea TFs aiding to the study of chickpea signalling geometrically. Furthermore, we developed few short webAPI(s) and several webinterfaces to facilitate advanced users to query the database for information in individual or bulk through single URL or incorporate it into other database pipelines. The CicerTransDB would provide a user-friendly interface for retrieving useful information specific to chickpea, which is otherwise lacking in the existing database systems. Integration of other database through one-click interface adds an example of future database systems for a new dimension of user-friendliness.

## Construction and content

This section describes briefly the process of recruiting TFs, making of the database, utility of the CicerTransDB webserver and usage of the webAPI/webinterfaces.

### Searching for chickpea TFs and generation of data

The annotation data used in this study was acquired from Chickpea Genome Annotation v 1.0 [1]. Protein sequences were analysed through pfam [11] in batch mode, and the retrieved data was parsed to tab format using perl scripts. The parsed data was fed into Mariadb for initial local database and queried for specific domains as specified in PlantTFDB [12], generating the primary list. The primary list thus obtained was manually examined and catalogued into various classes according to the classification system described earlier [9, 12]. Additionally, Tub domain, previously published from our lab [13], was added to this list finalising the secondary list (Additional file 1: Table S1). This was considered as the final TFlist, which was used as a template for obtaining additional data.

The TFlist was blasted against NCBInr database (updated Dec, 2014) for naming the genes, using blastp program with e-value 1e-03 and max 20 targets [14]. Additionally, custom perl scripts were used to retrieve 1.5 kb upstream promoter sequences from the chickpea genome [1]. The data was analyzed for *cis*-acting regulatory elements using PLACE database [15], and the output was converted to SQL table. We also developed a PLACE parser using perl to convert PLACE output into SQL compatible tab format (data not shown). The complete set of protein sequences were analyzed through LocTree 3.0 and LocTree 2.0 [16, 17] programs to predict their localization. Each TF sequence was searched against TAIR [18] and PlantTFDB for annotation of functions of chickpea TFs, via blastp using e-value 1e-03 and 1e-06, respectively, with identity cutoff of 80 %. The protein homologues were also searched against SwissProt [19] with an e-value of 1e-03 and cutoff identity of 80 %. The mapped SwissProt ID and TAIR ID were then employed to map Gene Ontology using map tables obtained through Gene Ontology Consortium [20]. The summary information from all these sources would offer a strong knowledge-based foundation for further characterization of chickpea TFs.

### Development of database and webserver

We used MariaDB as the data management system for the generation of primary and final database. The webserver is hosted at www.hostinger.in with online server database system to be MySQL. The dynamic pages and webAPI were built in PHP and Javascript.

### Website structure

The user interface of CicerTransDB was developed on framework of PHP. This enables the use of robust hidden layers which is secured from user, as well as diverse and dynamic front end layout that provides user with variety of information. A schematic diagram of the workflow is shown in Figure S1 of Additional file 2.

## Utility and discussion

### Website features

#### Browse data

**Fig. 1:**
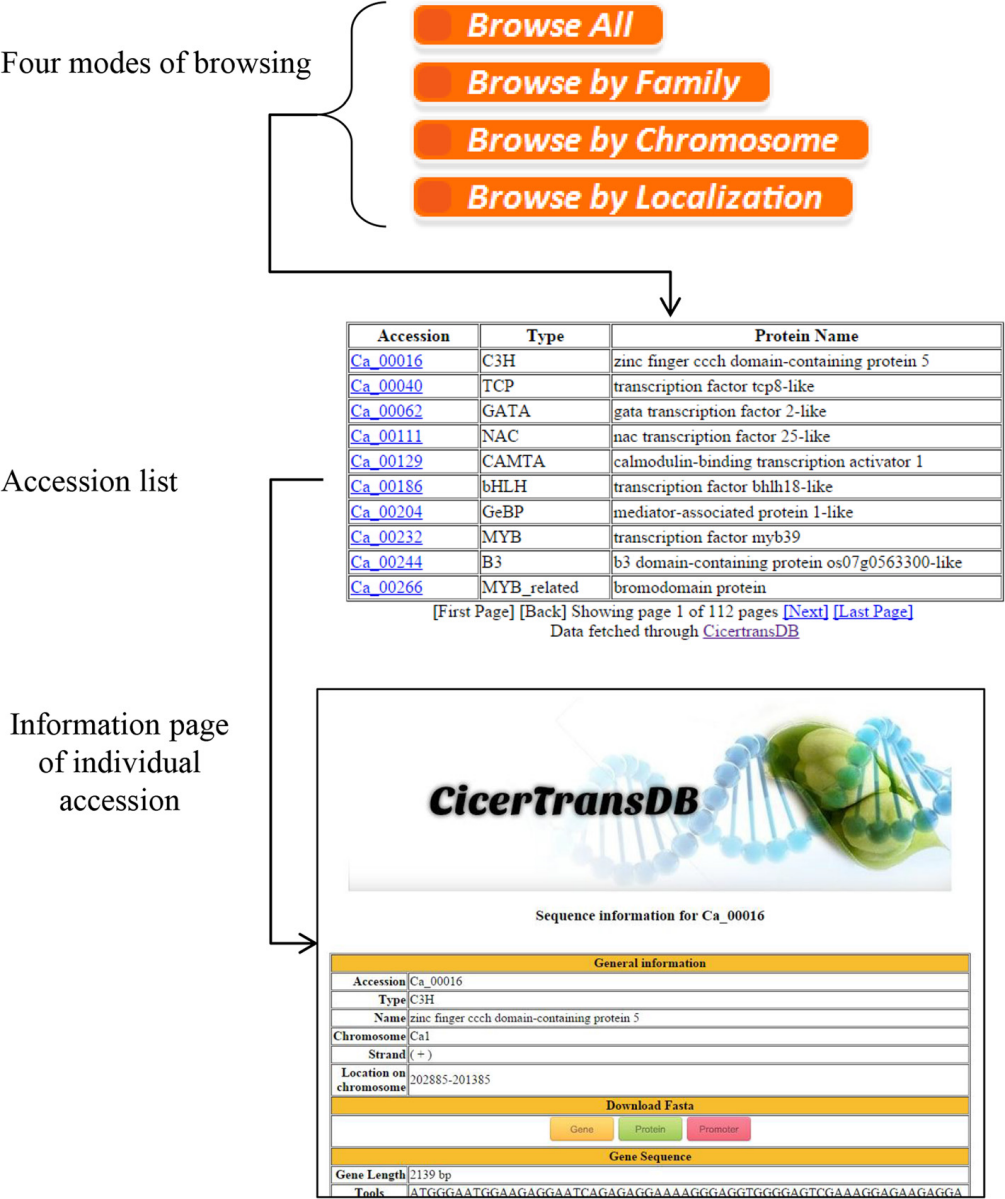
Workflow of browsing features of CicerTransDB

CicerTransDB provides user an option to browse data in four modes: all, by family, by chromosomes or by localization. The output is a list of accessions, each of which is linked to a detailed information page for that particular protein member. A brief screenshot is shown in Fig. 1.

#### Search form and diversity of query

**Fig. 2:**
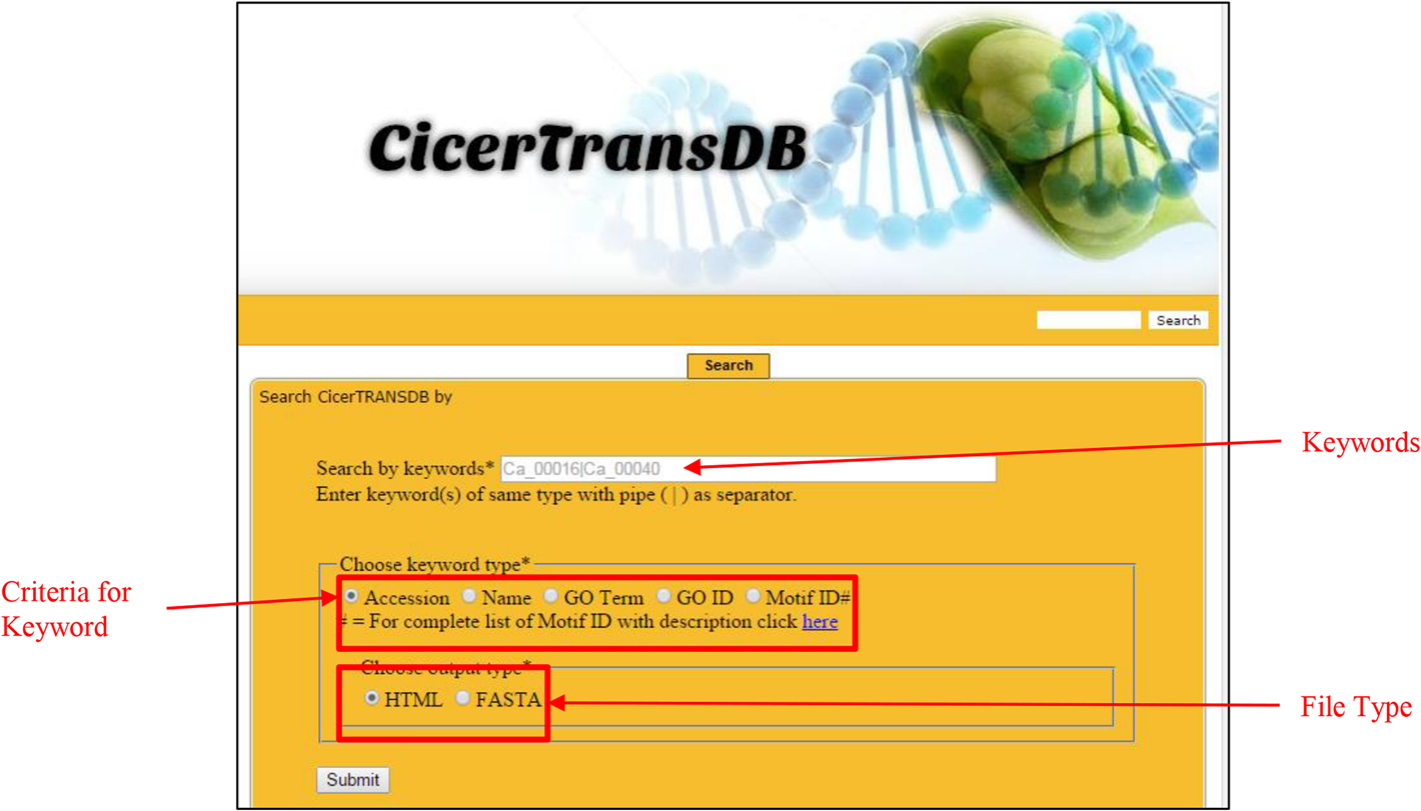
Screenshot of the search page with description of various sections

CicerTransDB can be searched in bulk mode for various criteria including accession, name, go term, go ID and motif ID. User can retrieve the data in HTML format (list) or in fasta format directly. Figure 2 shows a screenshot of the search page with description of various sections. The search form can be used in many ways. To demonstrate usefulness of the database, a demo page has been created at http://cicertransdb.esy.es/documents/demo.html. The page output shows a list of dehydration-responsive TFs in chickpea.

#### Download bulk data

CicerTransDB has the feature for downloading data in bulk which includes both TF sequences and feature tables. The sequences can be downloaded in either fasta, excel or tab delimited text format. The fasta headers are pipe (|) delimited containing information such as location, strand and name. The name is put on fifth position in accordance to NCBI blast convention facilitating parsing programs. The user has the option of downloading all sequences as well as sequences based on particular TF family, chromosome or localization. Furthermore, user can also download features such as accession list, list of motifs, list of domains and list of homologues based on TF family, chromosome or localization in excel as well as tab delimited text format. A short tutorial on the above is included in the wiki section of the database. The wiki section also includes know-how to download bulk data directly through URL.

#### Data information of individual genes and cross database tools

**Fig. 3:**
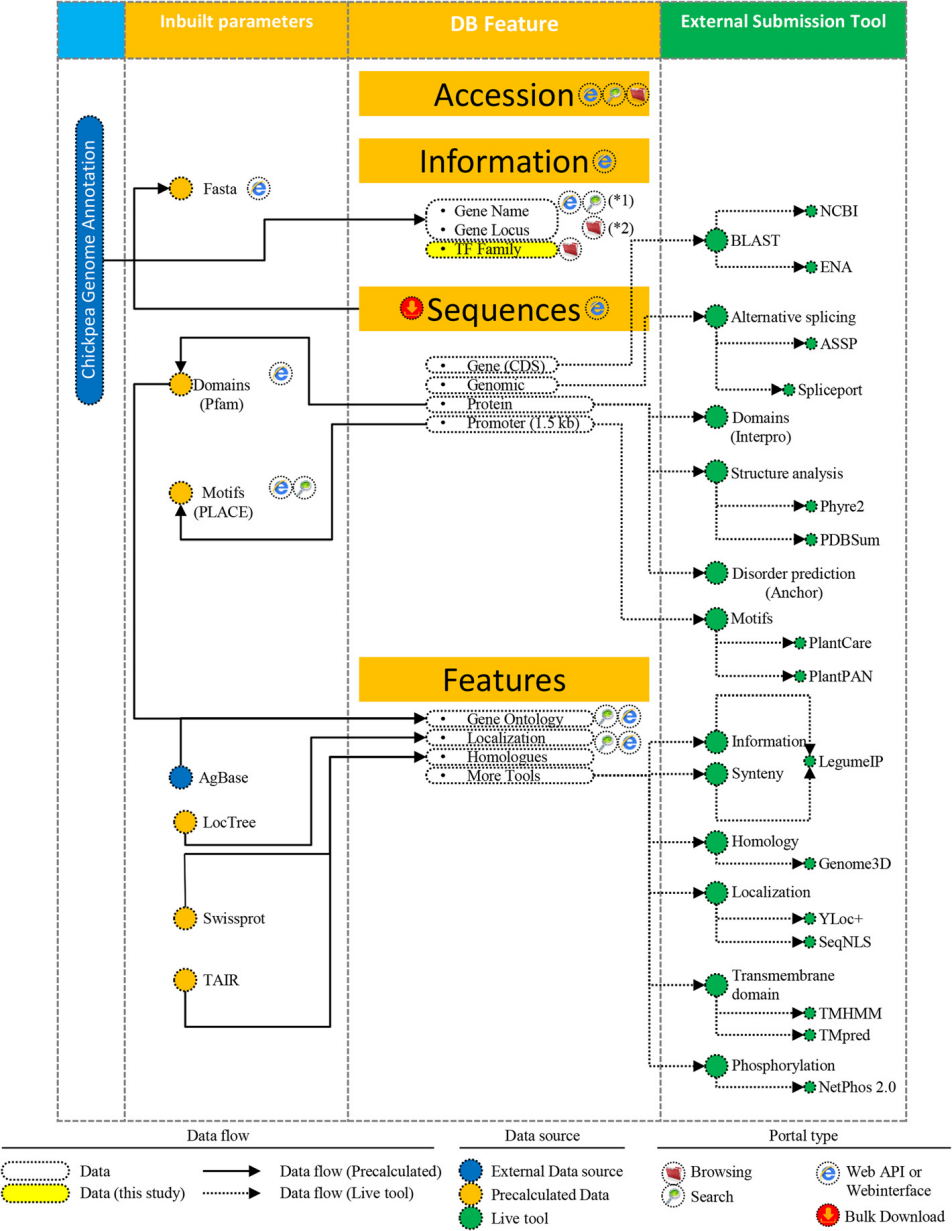
Schematic diagram showing various tools and dataflow of CicerTransDB. *1 denotes data obtained by blast. *2 denote data derived from annotation

CicerTransDB comprises information of 1124 TFs. The information incorporates data from genome annotation viz., chromosome, strand and location, protein, and gene sequence. Data comprising the name of protein, 1.5 kb upstream genomic sequence, features such as motifs (PLACE) and domains (Pfam), homologues from PlantTFDB and TAIR, Gene Ontology are also included. The website also holds many single-click interfaces to connect to other third party webservers that include motif analysis in PlantPAN [21] and PlantCare [22], gene blast at NCBI [14] and ENA [23], as well as localization prediction at Yloc + [24]. Furthermore, detailed information for motifs and domains and their position is also available. We have also designed a webinterface for motif and domain details of individual TF. A complete list is shown in schematic representation (Fig. 3) and the corresponding references are enlisted in ‘About’ section of the website.

#### WebAPI(s) and webinterface(s)

CicerTransDB integrates several APIs and interfaces, the first of which is for individual data that returns the information of accession number in html format. The interface further extends to information regarding fasta sequences, domain and motif details. For example, information of gene Ca_00040 can be obtained at www.cicertransdb.esy.es/data/Ca_00040, and the details of domains and motifs of Ca_00040 can be obtained at http://www.cicertransdb.esy.es/data/domain/Ca_00040 and http://www.cicertransdb.esy.es/data/motif/Ca_00040, respectively. All available features list is detailed in Fig. 3. The users can search and download the batch information using query in URL. A detailed explanation about how to formulate the URL is provided in the website’s wiki section.

### Data analysis

**Fig. 4:**
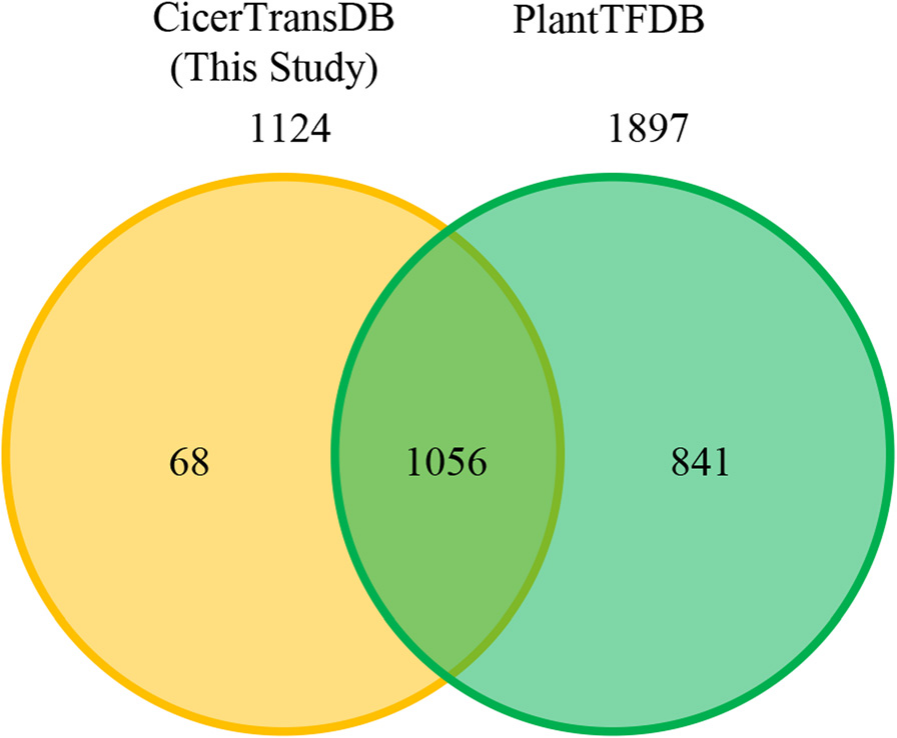
Venn diagram showing comparison of transcription factors in CicerTransDB with that in the PlantTFDB. The comparison reveals 68 new transcription factors in the chickpea genome which were previously unexplored

CicerTransDB is based on an annotation wherein a gene location harbors only a single protein and thus, would yield better information about evolution and diversity of TFs. The comparison of CicerTransDB and PlantTFDB revealed that both the databases contain unique sequences (Fig. 4). CicerTransDB comprises 68 unique TFs not previously reported in PlantTFDB, emphasizing the importance of genome-wide studies on recruitment of TFs.

We designed a new chromosome map utility based on PHP for making chromosome maps in CicerTransDB. Chromosome map for each TF family is accessible both through website as well as webAPI. The number of TFs and average TF density were calculated for each chromosome, which showed the lowest number of TFs in Ca2 and Ca8, although the average TF density per chromosome was highest on Ca8 (Figure S2 in Additional file 3). We also calculated the number of TFs per chromosome per family (Table S2 in Additional file 4). These data may facilitate in evolutionary studies of genes across related organisms.

### Future work

CicerTransDB is based on chickpea genome annotation v 1.0. We anticipate that future fine-tuning of the genome annotation and further release of new sequences would exert an extra pressure on release of new versions of CicerTransDB and maintaining data. Our future efforts will focus onto updating the database with new information as it becomes available. Deciphering new families of TFs through data-mining remains difficult, which will be an important task in upcoming versions of the database. CicerTransDB would predict direct binding targets of TFs throughout the chickpea genome.

## Conclusions

CicerTransDB is a cross platform database where user can query and view information about TFs of chickpea. It also provides advanced search tools, which can be used to generate diverse information from the database. Furthermore, the data can be queried though webAPI and webinterfaces to get data for individual TF as well as large datasets. Various third-party links have been provided as single-click interface to user for an easy workflow. Altogether, these features make CicerTransDB a unique and comprehensive resource for chickpea TF repertoire. Comparison of CicerTransDB with PlantTFDB revealed 68 unique TFs in chickpea genome, hitherto unexplored. The chromosome distributional analysis revealed that chickpea TFs are disseminated throughout its genome. In spite of having lowest number of TFs, chromosome Ca8 was found to have the highest average TF density among all chromosomes. We strongly believe that CicerTransDB will act as a conceptual framework for future computational and experimental research on the chickpea transcriptional regulatory network from the perspective of the TFs acting on cis-regulatory elements, and provide the necessary acceleration in this area of resaerch.

## Abbreviations

API, Application programming interface; GTFs, General transcription factors; HTML, HyperText Markup Language; PHP, Hypertext Preprocessor; PSI-BLAST, Position-Specific Iterated BLAST; SQL, Structured Query Language; TF, Transcription factor.

## Additional files

Additional file 1: Table S1. Manual curation of the primary list of transcription factors generated through domain search. Tubby* domain proteins are putative transcription factors (Wardhan et al. [13]), but not included in PlantTFDB. (PDF 1721 kb) Additional file 2: Figure S1. Diagram showing CicerTransDB webserver workflow. Bold red lines show user accessible pipeline. Blue and green dotted lines show workflow running through website PHP without user’s interaction. Blue lines are normal PHP data, while green lines are secured ones. (PDF 39 kb) Additional file 3: Figure S2. A bar chart showing distribution of transcription factors in chickpea genome on individual chromosomes and line chart showing average density of transcription factors in each chromosome. The data shows count of all TF types. Individual family distribution graph has been included in the database website. (PDF 26 kb) Additional file 4: Table S2. Distribution of TF family genes across the chromosomes. (PDF 42 kb)
