# Supplementary material for: CicerTransDB 1.0: a resource for expression and functional study of chickpea transcription factors
Source: BMC Plant Biol. 2016 Jul 29;16:169. doi: 10.1186/s12870-016-0860-y (PMC4966752; doi:10.1186/s12870-016-0860-y)
Supplement: Additional file 1: Table S1. — Manual curation of the primary list of transcription factors generated through domain search. Tubby* domain proteins are putative transcription factors (Wardhan et al. [13]), but not included in PlantTFDB. (PDF 1721 kb) [file 12870_2016_860_MOESM1_ESM.pdf]

Additional file 1: Table S1. Generation of secondary list by manual curation of the primary list of transcription factors generated through domain search. Tubby* domain proteins are putative transcription factors (Wardhan et al., 2012), but not included in PlantTFDB.

Accession	TF type	1	2	3	4	5
Ca_00007	#NA	PF00076				
Ca_00010	#NA	PF06507				
**Ca_00016**	**C3H**	PF00642	PF00642			
**Ca_00040**	**TCP**	PF03634				
Ca_00051	#NA	PF00271				
**Ca_00062**	**GATA**	PF00320				
Ca_00065	#NA	PF00271				
**Ca_00111**	**NAC**	PF02365				
**Ca_00129**	**CAMTA**	PF03859				
Ca_00133	#NA	PF00656				
Ca_00134	#NA	PF00656	PF00656			
**Ca_00186**	**bHLH**	PF00010				
**Ca_00204**	**GeBP**	PF04504				
Ca_00230	#NA	PF08879				
**Ca_00232**	**MYB**	PF00249	PF00249			
**Ca_00244**	**B3**	PF02362				
**Ca_00266**	**MYB_related**	PF00249				
Ca_00274	#NA	PF00271				
Ca_00285	#NA	PF00076				
**Ca_00295**	**Dof**	PF02701				
**Ca_00318**	**Dof**	PF02701				
**Ca_00319**	**Dof**	PF02701				
**Ca_00320**	**SBP**	PF03110				
**Ca_00324**	**C3H**	PF00642				
**Ca_00326**	**ERF**	PF00847				
**Ca_00327**	**ERF**	PF00847				
**Ca_00337**	**HB-other**	PF00046				
**Ca_00344**	**NAC**	PF02365				
**Ca_00347**	**ARF**	PF02362	PF06507			
**Ca_00357**	**HB-other**	PF00046				
**Ca_00359**	**ERF**	PF00847				
**Ca_00360**	**ERF**	PF00847				
Ca_00364	#NA	PF00271				
**Ca_00387**	**bHLH**	PF00010				
**Ca_00392**	**Dof**	PF02701				
**Ca_00417**	**ERF**	PF00847				
**Ca_00420**	**WRKY**	PF03106				
**Ca_00428**	**HSF**	PF00447				
**Ca_00445**	**MIKC**	PF00319	PF01486			
**Ca_00467**	**ARF**	PF02362	PF06507			
**Ca_00482**	**TCP**	PF03634				
Ca_00506	#NA	PF00076				
**Ca_00512**	**C3H**	PF00642	PF00642	PF00642	PF00642	
**Ca_00514**	**ERF**	PF00847				
Ca_00523	#NA	PF00271				
**Ca_00550**	**SBP**	PF03110				
Ca_00593	#NA	PF00076	PF00076	PF00076		
**Ca_00615**	**bHLH**	PF00010				

Accession	TF type	1	2	3	4	5
**Ca_00645**	**MYB_related**	PF00249				
**Ca_00656**	**WRKY**	PF03106				
Ca_00668	#NA	PF03789				
**Ca_00703**	**MYB**	PF00249	PF00249			
**Ca_00729**	**M-type**	PF00319				
Ca_00732	#NA	PF00628				
Ca_00740	#NA	PF00076	PF00076	PF00076	PF00076	
**Ca_00750**	**HB-other**	PF00046				
**Ca_00762**	**C3H**	PF00642				
**Ca_00768**	**ERF**	PF00847				
**Ca_00772**	**GRF**	PF08880	PF08879			
**Ca_00780**	**bZIP_1**	PF00170				
**Ca_00791**	**bZIP_1**	PF00170				
Ca_00806	#NA	PF00076	PF00076	PF00076		
**Ca_00818**	**NAC**	PF02365				
Ca_00822	#NA	PF00076				
**Ca_00826**	**B3**	PF02362				
Ca_00865	#NA	PF00076	PF00076			
**Ca_00866**	**NF-YA**	PF02045				
Ca_00916	#NA	PF00076	PF00076	PF00076		
**Ca_00927**	**CO-like**	PF00643	PF00643	PF06203		
**Ca_00943**	**C2H2**	PF00096	PF00096			
Ca_00964	#NA	PF00076	PF00076			
Ca_00968	#NA	PF00076				
**Ca_00990**	**C3H**	PF00642				
**Ca_01004**	**bZIP_1**	PF00170				
**Ca_01009**	**GRAS**	PF03514				
**Ca_01010**	**WRKY**	PF03106				
Ca_01014	#NA	PF00271				
**Ca_01018**	**WRKY**	PF03106				
Ca_01019	#NA	PF00076	PF00076			
**Ca_01036**	**SBP**	PF03110				
**Ca_01050**	**WRKY**	PF03106				
**Ca_01053**	**bHLH**	PF00010				
**Ca_01055**	**MYB**	PF00249	PF00249			
Ca_01066	#NA	PF00271				
**Ca_01072**	**MYB**	PF00249	PF00249			
**Ca_01111**	**ERF**	PF00847				
**Ca_01115**	**GATA**	PF00320				
Ca_01116	#NA	PF00929				
Ca_01118	#NA	PF00628				
**Ca_01181**	**bHLH**	PF00010				
**Ca_01216**	**GeBP**	PF04504				
**Ca_01234**	**bZIP_1**	PF00170				
**Ca_01256**	**MYB_related**	PF00249				
**Ca_01263**	**MYB_related**	PF00249				
**Ca_01293**	**bZIP_1**	PF00170				
Ca_01311	#NA	PF00628				

Accession	TF type	1	2	3	4	5
**Ca_01324**	**bHLH**	PF00010				
**Ca_01325**	**ERF**	PF00847				
**Ca_01329**	**MYB**	PF00249	PF00249			
**Ca_01331**	**Dof**	PF02701				
Ca_01349	#NA	PF00076	PF00076			
Ca_01357	#NA	PF00076	PF00076			
Ca_01359	#NA	PF00072	PF06203			
Ca_01367	#NA	PF00271				
**Ca_01368**	**B3**	PF02362				
Ca_01369	#NA	PF00076				
**Ca_01386**	**LFY**	PF01698				
**Ca_01387**	**ERF**	PF00847				
Ca_01390	#NA	PF00076	PF00076			
**Ca_01394**	**bHLH**	PF00010				
**Ca_01402**	**bHLH**	PF00010				
**Ca_01414**	**NAC**	PF02365				
Ca_01422	#NA	PF04433	PF00249			
**Ca_01426**	**SBP**	PF03110				
Ca_01450	#NA	PF00076				
**Ca_01453**	**bHLH**	PF00010				
Ca_01470	GATA (New)	PF06203	PF00320			
Ca_01471	GATA (New)	PF06203	PF00320			
**Ca_01481**	**MYB_related**	PF00249				
**Ca_01491**	**M-type**	PF00319				
**Ca_01503**	**ZF-HD**	PF04770				
**Ca_01522**	**MYB**	PF00249	PF00249			
Ca_01529	#NA	PF00076	PF00076			
**Ca_01536**	**B3**	PF02362				
**Ca_01548**	**bZIP_1**	PF00170				
**Ca_01550**	**bHLH**	PF00010				
**Ca_01551**	**HSF**	PF00447				
Ca_01555	#NA	PF00076	PF00076	PF00076	PF00076	
**Ca_01556**	**ERF**	PF00847				
**Ca_01572**	**M-type**	PF00319				
**Ca_01588**	**bHLH**	PF00010				
Ca_01594	#NA	PF00628				
Ca_01609	#NA	PF00076	PF00076			
**Ca_01610**	**AP2**	PF00847	PF00847			
**Ca_01617**	**HB-other**	PF00046				
**Ca_01625**	**bHLH**	PF00010				
**Ca_01640**	**HSF**	PF00447				
Ca_01645	#NA	PF00076				
**Ca_01656**	**Nin-like**	PF02042				
Ca_01677	#NA	PF00271				
Ca_01679	#NA	PF00076				
**Ca_01683**	**ERF**	PF00847				
Ca_01688	#NA	PF00072				
**Ca_01694**	**CO-like**	PF00643	PF00643	PF06203		

Accession	TF type	1	2	3	4	5
**Ca_01713**	**WRKY**	PF03106				
Ca_01723	#NA	PF00628	PF00271			
Ca_01726	#NA	PF00072				
**Ca_01729**	**ERF**	PF00847				
Ca_01733	#NA	PF03789				
**Ca_01764**	**ERF**	PF00847				
Ca_01768	#NA	PF00271				
**Ca_01772**	**HB-other**	PF00046				
**Ca_01844**	**WRKY**	PF03106				
Ca_01847	#NA	PF00076				
Ca_01851	#NA	PF00076	PF00076	PF00076		
**Ca_01865**	**bZIP_1**	PF00170				
**Ca_01884**	**MYB**	PF00249	PF00249	PF00249		
**Ca_01892**	**CPP**	PF03638	PF03638			
**Ca_01908**	**ERF**	PF00847				
**Ca_01911**	**MYB_related**	PF00249				
**Ca_01912**	**MYB_related**	PF00249				
**Ca_01918**	**GATA**	PF00320				
**Ca_01919**	**HD-ZIP**	PF00046	PF01852			
Ca_01925	#NA	PF00076				
Ca_01956	#NA	PF00072				
**Ca_01974**	**HB-other**	PF00046				
**Ca_01981**	**NAC**	PF02365				
Ca_01985	#NA	PF00643	PF00643			
**Ca_01992**	**GATA**	PF00320				
**Ca_01994**	**MIKC**	PF00319	PF01486			
**Ca_02004**	**SRS**	PF05142				
**Ca_02034**	**ZF-HD**	PF04770				
**Ca_02038**	**GATA**	PF00320				
Ca_02052	#NA	PF00271	PF00271			
**Ca_02070**	**HB-other**	PF00046				
**Ca_02075**	**BES1**	PF05687				
**Ca_02122**	**MYB_related**	PF00249				
**Ca_02124**	**LBD (AS2/LOB)**	PF03195				
**Ca_02125**	**LBD (AS2/LOB)**	PF03195				
**Ca_02146**	**HSF**	PF00447				
Ca_02152	#NA	PF00076	PF00076	PF00076		
**Ca_02154**	**bHLH**	PF00010				
**Ca_02162**	**bHLH**	PF00010				
**Ca_02168**	**AP2**	PF00847	PF00847	PF00847		
**Ca_02170**	**ERF**	PF00847				
Ca_02171	#NA	PF00076	PF00076			
**Ca_02208**	**LBD (AS2/LOB)**	PF03195				
**Ca_02226**	**GRAS**	PF03514				
**Ca_02232**	**MYB_related**	PF00249				
**Ca_02245**	**AP2**	PF00847	PF00847			
**Ca_02249**	**MYB**	PF00249	PF00249			
**Ca_02251**	**HB-other**	PF00046				

Accession	TF type	1	2	3	4	5
Ca_02262	bHLH	PF00010				
Ca_02263	bHLH	PF00010				
Ca_02264	bHLH	PF00010				
Ca_02274	NAC	PF02365				
Ca_02285	MYB_related (New)	PF00072	PF00249			
Ca_02313	GATA	PF00320				
Ca_02325	ERF	PF00847				
Ca_02329	HB-other	PF00046				
Ca_02365	NAC	PF02365				
Ca_02401	#NA	PF03789				
Ca_02407	ERF	PF00847				
Ca_02408	ERF	PF00847				
Ca_02416	HSF	PF00447				
Ca_02417	MYB_related	PF00249				
Ca_02437	#NA	PF06203				
Ca_02450	#NA	PF00076				
Ca_02452	MYB_related	PF00249				
Ca_02457	NAC	PF02365				
Ca_02459	C2H2	PF00096				
Ca_02473	TCP	PF03634				
Ca_02482	bHLH	PF00010				
Ca_02499	AP2	PF00847	PF00847			
Ca_02507	LBD (AS2/LOB)	PF03195				
Ca_02510	Dof	PF02701				
Ca_02516	ARF	PF02362	PF06507			
Ca_02520	NAC	PF02365				
Ca_02539	HD-ZIP	PF00046	PF01852			
Ca_02541	ARF	PF02362	PF06507			
Ca_02542	GATA	PF00320				
Ca_02543	#NA	PF01852				
Ca_02553	#NA	PF00271				
Ca_02579	#NA	PF06203				
Ca_02586	#NA	PF00076				
Ca_02593	MYB	PF00249	PF00249			
Ca_02608	Dof	PF02701				
Ca_02625	GRAS	PF03514				
Ca_02626	GRAS	PF03514				
Ca_02645	#NA	PF00076	PF00076	PF00076		
Ca_02657	AP2	PF00847	PF00847			
Ca_02661	FAR1	PF03101				
Ca_02662	bHLH	PF00010				
Ca_02669	EIL	PF04873				
Ca_02686	FAR1	PF03101				
Ca_02689	#NA	PF00643				
Ca_02692	bZIP_1	PF00170				
Ca_02696	GRAS	PF03514				
Ca_02703	Nin-like	PF02042				
Ca_02713	NF-YA	PF02045				

Accession	TF type	1	2	3	4	5
Ca_02714	#NA	PF00628				
**Ca_02722**	**MYB_related**	PF00249				
Ca_02724	#NA	PF00271				
**Ca_02730**	**MYB**	PF00249	PF00249			
**Ca_02741**	**LBD (AS2/LOB)**	PF03195				
Ca_02750	#NA	PF00271				
Ca_02756	#NA	PF00929				
**Ca_02767**	**bHLH**	PF00010				
**Ca_02786**	**MYB**	PF00249				
**Ca_02790**	**HD-ZIP**	PF00046	PF01852			
**Ca_02812**	**HD-ZIP**	PF00046	PF01852			
**Ca_02862**	**C3H**	PF00642	PF00642			
**Ca_02880**	**ERF**	PF00847				
**Ca_02910**	**bHLH**	PF00010				
**Ca_02911**	**bHLH**	PF00010				
**Ca_02925**	**WRKY**	PF03106				
Ca_02989	MYB_related (New)	PF00072	PF00249			
**Ca_02992**	**GATA**	PF00320				
**Ca_02997**	**CAMTA**	PF03859				
**Ca_03027**	**WRKY**	PF03106				
Ca_03031	#NA	PF00076	PF00076			
**Ca_03053**	**MYB**	PF00249	PF00249			
Ca_03085	#NA	PF00271				
**Ca_03128**	**ARF**	PF02362	PF06507			
Ca_03132	#NA	PF00076	PF00076			
**Ca_03154**	**MYB**	PF00249	PF00249			
Ca_03177	#NA	PF00271				
**Ca_03183**	**HSF**	PF00447				
**Ca_03193**	**GATA**	PF00320				
**Ca_03200**	**HB-other**	PF00046				
**Ca_03206**	**WRKY**	PF03106				
Ca_03219	#NA	PF06203				
**Ca_03239**	**AP2**	PF00847	PF00847			
**Ca_03266**	**MYB**	PF00249	PF00249			
**Ca_03275**	**CO-like**	PF00643	PF06203			
**Ca_03306**	**AP2**	PF00847	PF00847			
**Ca_03331**	**WRKY**	PF03106	PF03106			
**Ca_03340**	**bHLH**	PF00010				
Ca_03351	MYB_related (New)	PF00072	PF00249			
Ca_03356	#NA	PF00271				
Ca_03367	#NA	PF00076	PF00076	PF00076		
**Ca_03388**	**WRKY**	PF03106				
**Ca_03436**	**C3H**	PF00642	PF00642	PF00642	PF00642	
**Ca_03481**	**ERF**	PF00847				
**Ca_03482**	**ERF**	PF00847				
**Ca_03498**	**MYB**	PF00249	PF00249			
Ca_03503	#NA	PF00628				
Ca_03509	#NA	PF00072				

Accession	TF type	1	2	3	4	5
**Ca_03512**	**bZIP_1**	PF0017				
**Ca_03524**	**WRKY**	PF0310				
Ca_03530	#NA	PF0027				
**Ca_03535**	**MYB**	PF0024	PF0024			
**Ca_03536**	**MYB**	PF0024	PF0024			
**Ca_03548**	**SBP**	PF0311				
**Ca_03564**	**MYB**	PF0024	PF0024			
Ca_03580	#NA	PF0027				
**Ca_03589**	**MYB**	PF0024	PF0024			
**Ca_03591**	**bHLH**	PF0001				
**Ca_03592**	**M-**	PF0031				
Ca_03625	#NA	PF0378				
Ca_03638	#NA	PF0027				
Ca_03663	#NA	PF0007	PF0007			
**Ca_03665**	**ERF**	PF0084				
Ca_03672	#NA	PF0027				
Ca_03675	#NA	PF0027	PF0027			
Ca_03676	#NA	PF0887				
**Ca_03678**	**C2H2**	PF0009				
**Ca_03681**	**MYB_related**	PF0024				
**Ca_03703**	**bHLH**	PF0001				
**Ca_03737**	**GeBP**	PF0450				
**Ca_03738**	**GeBP**	PF0450				
**Ca_03753**	**bZIP_1**	PF0017				
**Ca_03755**	**bHLH**	PF0001				
Ca_03778	#NA	PF0027				
**Ca_03781**	**MYB_related**	PF0024				
Ca_03787	#NA	PF0007				
Ca_03800	#NA	PF0007				
**Ca_03824**	**C3H**	PF0064	PF0064	PF0064	PF0064	PF0064
**Ca_03825**	**WRKY**	PF0310				
Ca_03827	#NA	PF0007	PF0007			
Ca_03833	#NA	PF0185				
**Ca_03849**	**Dof**	PF0270				
**Ca_03887**	**MIKC**	PF0031	PF0148			
**Ca_03888**	**MYB_related**	PF0024				
**Ca_03906**	**MYB_related**	PF0024				
**Ca_03920**	**GRAS**	PF0351				
**Ca_03948**	**ERF**	PF0084				
**Ca_03951**	**bHLH**	PF0001				
**Ca_03952**	**WRKY**	PF0310				
Ca_03974	#NA	PF0620				
**Ca_03992**	**Nin-like**	PF0204				
**Ca_04005**	**Dof**	PF0270				
Ca_04007	#NA	PF0007				
**Ca_04027**	**HB-other**	PF0004				
**Ca_04035**	**GRF**	PF0888	PF0887			
Ca_04041	#NA	PF0007				

Accession	TF type	1	2	3	4	5
Ca_04042	#NA	PF00076	PF00076			
Ca_04043	#NA	PF00076	PF00076			
Ca_04044	#NA	PF00076	PF00076			
Ca_04056	#NA	PF00072	PF06203			
**Ca_04069**	**NAC**	PF02365				
**Ca_04100**	**WRKY**	PF03106				
Ca_04102	#NA	PF00072				
**Ca_04108**	**bZIP_1**	PF00170				
**Ca_04126**	**LBD (AS2/LOB)**	PF03195				
**Ca_04141**	**WRKY**	PF03106				
**Ca_04144**	**WRKY**	PF03106				
**Ca_04147**	**ERF**	PF00847				
Ca_04149	#NA	PF00076	PF00076			
Ca_04175	#NA	PF00628				
**Ca_04178**	**C3H**	PF00642	PF00642			
**Ca_04187**	**NAC**	PF02365				
**Ca_04233**	**NAC**	PF02365				
**Ca_04277**	**MYB_related**	PF00249				
Ca_04285	#NA	PF00271				
**Ca_04287**	**LBD (AS2/LOB)**	PF03195				
Ca_04295	#NA	PF00076				
**Ca_04298**	**GRAS**	PF03514				
**Ca_04300**	**C2H2**	PF00096				
**Ca_04309**	**NAC**	PF02365				
Ca_04336	#NA	PF00076				
**Ca_04337**	**NAC**	PF02365				
Ca_04344	#NA	PF00076	PF00076	PF00076	PF00076	
**Ca_04370**	**ERF**	PF00847				
Ca_04372	#NA	PF00076				
**Ca_04375**	**bZIP_1**	PF00170				
Ca_04399	#NA	PF00076				
**Ca_04423**	**WRKY**	PF03106				
**Ca_04428**	**S1Fa-like**	PF04689				
**Ca_04442**	**GRAS**	PF03514				
**Ca_04456**	**Nin-like**	PF02042				
**Ca_04477**	**MYB**	PF00249	PF00249			
**Ca_04488**	**HSF**	PF00447				
**Ca_04491**	**HD-ZIP**	PF00046	PF01852			
Ca_04500	#NA	PF00076	PF00076			
**Ca_04503**	**ERF**	PF00847				
**Ca_04504**	**ERF**	PF00847				
**Ca_04518**	**MIKC**	PF00319	PF01486			
Ca_04535	#NA	PF00271				
**Ca_04554**	**HSF**	PF00447				
Ca_04596	#NA	PF00628				
Ca_04622	#NA	PF00271				
**Ca_04629**	**HD-ZIP**	PF00046	PF01852			
**Ca_04658**	**M-type**	PF00319				

Accession	TF type	1	2	3	4	5
**Ca_04659**	**M-type**	PF00319				
**Ca_04660**	**M-type**	PF00319				
**Ca_04661**	**M-type**	PF00319				
**Ca_04662**	**M-type**	PF00319				
**Ca_04665**	**M-type**	PF00319				
Ca_04691	#NA	PF00628				
Ca_04700	#NA	PF00271				
**Ca_04722**	**MYB_related**	PF00249				
**Ca_04754**	**GATA**	PF00320				
Ca_04758	#NA	PF00643	PF00643			
Ca_04760	#NA	PF00628				
**Ca_04767**	**bZIP_1**	PF00170				
**Ca_04778**	**bHLH**	PF00010				
**Ca_04779**	**MYB_related**	PF00249				
Ca_04780	#NA	PF00076				
Ca_04800	#NA	PF00076				
Ca_04801	#NA	PF04433	PF00249			
**Ca_04804**	**NAC**	PF02365				
**Ca_04823**	**HB-other**	PF00046				
**Ca_04827**	**ARF**	PF02362	PF06507			
**Ca_04834**	**Nin-like**	PF02042				
**Ca_04880**	**MYB_related**	PF00249				
**Ca_04894**	**E2F/DP**	PF02319				
**Ca_04909**	**MIKC**	PF00319	PF01486			
**Ca_04946**	**MYB_related**	PF00249				
**Ca_04960**	**C2H2**	PF00096				
**Ca_04963**	**BES1**	PF05687				
**Ca_04981**	**bHLH**	PF00010				
**Ca_05023**	**M-type**	PF00319				
**Ca_05025**	**ARF**	PF02362	PF06507			
Ca_05029	#NA	PF00271				
**Ca_05033**	**TCP**	PF03634				
**Ca_05046**	**GRAS**	PF03514				
**Ca_05083**	**C2H2**	PF00096	PF00096			
**Ca_05097**	**C3H**	PF00642				
Ca_05116	#NA	PF01852				
**Ca_05117**	**bHLH**	PF00010				
**Ca_05118**	**bHLH**	PF00010				
Ca_05139	#NA	PF00628	PF00271	PF00271		
**Ca_05173**	**WRKY**	PF03106				
**Ca_05204**	**WRKY**	PF03106				
**Ca_05211**	**bZIP_1**	PF00170				
Ca_05223	#NA	PF00076				
**Ca_05227**	**NAC**	PF02365				
**Ca_05231**	**C3H**	PF00642				
Ca_05248	#NA	PF00076				
**Ca_05255**	**CAMTA**	PF03859				
Ca_05258	#NA	PF06943	PF00656			

Accession	TF type	1	2	3	4	5
Ca_05259	#NA	PF00656				
Ca_05263	#NA	PF00076				
Ca_05292	GATA (New)	PF06203	PF00320			
**Ca_05320**	**bHLH**	PF00010				
Ca_05352	#NA	PF00271				
Ca_05408	#NA	PF00076				
**Ca_05416**	**bHLH**	PF00010				
**Ca_05422**	**MYB_related**	PF00249				
**Ca_05430**	**C2H2**	PF00096				
**Ca_05454**	**MYB**	PF00249	PF00249			
Ca_05466	#NA	PF00076				
**Ca_05471**	**bZIP_1**	PF00170				
**Ca_05485**	**ERF**	PF00847				
**Ca_05487**	**CO-like**	PF00643	PF06203			
**Ca_05488**	**LBD (AS2/LOB)**	PF03195				
**Ca_05503**	**MYB**	PF00249	PF00249			
**Ca_05547**	**C3H**	PF00642				
Ca_05551	#NA	PF00271				
Ca_05577	#NA	PF00076				
**Ca_05621**	**HB-PHD**	PF00628	PF00046			
**Ca_05643**	**bHLH**	PF00010				
**Ca_05644**	**bHLH**	PF00010				
**Ca_05681**	**ARF**	PF02362	PF06507			
**Ca_05688**	**bHLH**	PF00010				
**Ca_05693**	**BBR-BPC**	PF06217				
**Ca_05696**	**NAC**	PF02365				
**Ca_05711**	**SBP**	PF03110				
Ca_05723	#NA	PF00271				
**Ca_05750**	**bHLH**	PF00010				
Ca_05768	#NA	PF00271				
Ca_05769	#NA	PF06203				
**Ca_05777**	**MYB**	PF00249	PF00249			
**Ca_05782**	**WRKY**	PF03106				
**Ca_05803**	**ERF**	PF00847				
**Ca_05815**	**WRKY**	PF03106				
Ca_05836	#NA	PF00628				
Ca_05875	#NA	PF00271				
**Ca_05876**	**ARF**	PF02362	PF06507			
**Ca_05889**	**MYB_related**	PF00249				
**Ca_05907**	**C3H**	PF00642				
**Ca_05913**	**NAC**	PF02365				
**Ca_05941**	**ERF**	PF00847				
**Ca_05961**	**CPP**	PF03638	PF03638			
**Ca_05976**	**B3**	PF02362				
Ca_05985	#NA	PF06203				
**Ca_05989**	**NAC**	PF02365				
Ca_06030	#NA	PF00076				
**Ca_06032**	**ERF**	PF00847				

Accession	TF type	1	2	3	4	5
**Ca_06034**	**ERF**	PF00847				
Ca_06065	#NA	PF00076	PF00076	PF00076		
**Ca_06085**	**bHLH**	PF00010				
**Ca_06099**	**AP2**	PF00847	PF00847			
**Ca_06124**	**WRKY**	PF03106	PF03106			
**Ca_06133**	**C3H**	PF00642	PF00642	PF00642	PF00642	PF00642
**Ca_06163**	**WRKY**	PF03106				
**Ca_06167**	**WRKY**	PF03106				
Ca_06189	#NA	PF00271				
**Ca_06210**	**BES1**	PF05687				
Ca_06225	#NA	PF00271				
**Ca_06226**	**MYB**	PF00249	PF00249			
**Ca_06267**	**HD-ZIP**	PF00046	PF01852			
Ca_06279	#NA	PF01486				
**Ca_06280**	**MIKC**	PF00319	PF01486			
**Ca_06308**	**LSD**	PF06943	PF06943			
**Ca_06323**	**B3**	PF02362				
**Ca_06326**	**Dof**	PF02701				
**Ca_06343**	**HB-other**	PF00046				
**Ca_06348**	**NAC**	PF02365				
**Ca_06352**	**ARF**	PF02362	PF06507			
**Ca_06365**	**HB-other**	PF00046				
**Ca_06368**	**LBD (AS2/LOB)**	PF03195				
Ca_06385	#NA	PF00076	PF00076			
**Ca_06386**	**bHLH**	PF00010				
**Ca_06422**	**MYB_related**	PF00249				
Ca_06423	#NA	PF00076				
Ca_06431	#NA	PF00076	PF00076			
**Ca_06446**	**ERF**	PF00847				
**Ca_06458**	**bZIP_1**	PF00170				
**Ca_06466**	**bHLH**	PF00010				
Ca_06476	#NA	PF00076	PF00076	PF00076	PF00076	
**Ca_06495**	**M-type**	PF00319				
**Ca_06496**	**HB-other**	PF00046				
**Ca_06508**	**HB-other**	PF00046				
**Ca_06548**	**LBD (AS2/LOB)**	PF03195				
**Ca_06552**	**B3**	PF02362				
**Ca_06590**	**ERF**	PF00847				
Ca_06600	#NA	PF00271				
**Ca_06609**	**TCP**	PF03634				
**Ca_06612**	**MYB**	PF00249	PF00249			
**Ca_06627**	**MYB_related**	PF00249				
Ca_06628	#NA	PF00076				
**Ca_06649**	**C3H**	PF00642	PF00642	PF00642		
Ca_06658	#NA	PF00271				
**Ca_06673**	**ERF**	PF00847				
**Ca_06681**	**YABBY**	PF04690				
Ca_06754	#NA	PF03789				

Accession	TF type	1	2	3	4	5
Ca_06761	#NA	PF00271				
Ca_06778	#NA	PF00076				
Ca_06811	#NA	PF00076				
**Ca_06823**	**bHLH**	PF00010				
Ca_06826	#NA	PF00271				
**Ca_06835**	**ERF**	PF00847				
**Ca_06836**	**C3H**	PF00642	PF00642	PF00642	PF00642	
**Ca_06837**	**MYB_related**	PF00249				
**Ca_06899**	**NAC**	PF02365				
**Ca_06901**	**NAC**	PF02365				
**Ca_06925**	**MYB_related**	PF00249				
**Ca_06929**	**NZZ/SPL**	PF08744				
**Ca_06943**	**GRAS**	PF03514				
**Ca_06956**	**bHLH**	PF00010				
**Ca_06984**	**WRKY**	PF03106				
**Ca_06993**	**MYB_related**	PF00249				
**Ca_07000**	**C2H2**	PF00096				
Ca_07002	#NA	PF00628				
Ca_07020	#NA	PF00628				
Ca_07021	#NA	PF00271				
**Ca_07023**	**C3H**	PF00642	PF00642			
**Ca_07056**	**bZIP_1**	PF00170				
**Ca_07057**	**bZIP_1**	PF00170				
**Ca_07058**	**MYB_related**	PF00249				
**Ca_07077**	**NAC**	PF02365				
**Ca_07101**	**YABBY**	PF04690				
**Ca_07113**	**WRKY**	PF03106				
**Ca_07119**	**MYB**	PF00249	PF00249			
**Ca_07131**	**HD-ZIP**	PF00046	PF01852			
**Ca_07144**	**MYB_related**	PF00249				
Ca_07152	#NA	PF00271				
**Ca_07159**	**bHLH**	PF00010				
**Ca_07170**	**bZIP_1**	PF00170				
Ca_07192	#NA	PF00072				
**Ca_07196**	**bHLH**	PF00010				
Ca_07218	#NA	PF00271				
**Ca_07234**	**HSF**	PF00447				
**Ca_07237**	**ERF**	PF00847				
**Ca_07252**	**C2H2**	PF00096				
**Ca_07261**	**C3H**	PF00642				
**Ca_07264**	**ERF**	PF00847				
**Ca_07284**	**bHLH**	PF00010				
**Ca_07292**	**bZIP_1**	PF00170				
**Ca_07299**	**SBP**	PF03110				
Ca_07302	#NA	PF00076	PF00076			
**Ca_07303**	**MYB_related**	PF00249				
**Ca_07304**	**MYB_related**	PF00249				
**Ca_07307**	**C3H**	PF00642	PF00642			

Accession	TF type	1	2	3	4	5
Ca_07320	#NA	PF08879				
**Ca_07322**	**HB-other**	PF00046				
Ca_07366	#NA	PF00076	PF00076	PF00076	PF00076	PF00076
Ca_07388	#NA	PF00271				
**Ca_07410**	**B3**	PF02362	PF02362			
**Ca_07413**	**C3H**	PF00642	PF00642	PF00642	PF00642	PF00642
**Ca_07414**	**C2H2**	PF00096				
Ca_07430	#NA	PF00076	PF00076			
Ca_07457	#NA	PF00076	PF00076			
**Ca_07460**	**MYB**	PF00249	PF00249			
**Ca_07463**	**LBD (AS2/LOB)**	PF03195				
Ca_07484	#NA	PF01852	PF01852			
**Ca_07488**	**Dof**	PF02701				
Ca_07527	#NA	PF00072				
**Ca_07534**	**EIL**	PF04873				
Ca_07544	#NA	PF00076				
Ca_07547	#NA	PF00076				
**Ca_07579**	**HB-other**	PF00046				
Ca_07582	#NA	PF00076				
**Ca_07607**	**M-type**	PF00319				
**Ca_07633**	**MYB_related**	PF00249				
**Ca_07641**	**CO-like**	PF00643	PF06203			
Ca_07643	#NA	PF00076	PF00076			
Ca_07647	#NA	PF00076				
Ca_07678	#NA	PF00628				
**Ca_07691**	**bHLH**	PF00010				
**Ca_07735**	**GRAS**	PF03514				
Ca_07738	#NA	PF00271				
**Ca_07743**	**NAC**	PF02365				
**Ca_07754**	**ERF**	PF00847				
**Ca_07755**	**ERF**	PF00847				
**Ca_07763**	**LBD (AS2/LOB)**	PF03195				
**Ca_07779**	**WRKY**	PF03106				
**Ca_07790**	**bHLH**	PF00010				
**Ca_07800**	**HSF**	PF00447				
**Ca_07836**	**bHLH**	PF00010				
**Ca_07844**	**bHLH**	PF00010				
Ca_07867	#NA	PF00076				
**Ca_07872**	**MYB**	PF00249	PF00249			
Ca_07884	#NA	PF00076	PF00076			
**Ca_07887**	**GRAS**	PF03514				
Ca_07892	#NA	PF00249	PF04433			
**Ca_07896**	**FAR1**	PF03101				
**Ca_07938**	**MYB_related**	PF00249				
**Ca_07939**	**MYB_related**	PF00249				
**Ca_07960**	**FAR1**	PF03101				
**Ca_07994**	**HD-ZIP**	PF00046	PF01852			
**Ca_07995**	**NF-YA**	PF02045				

Accession	TF type	1	2	3	4	5
Ca_08007	#NA	PF00271				
Ca_08013	#NA	PF01852				
**Ca_08014**	**HD-ZIP**	PF00046	PF01852			
**Ca_08015**	**HD-ZIP**	PF00046	PF01852			
**Ca_08016**	**HD-ZIP**	PF00046	PF01852			
**Ca_08043**	**MYB_related**	PF00249				
**Ca_08049**	**WRKY**	PF03106				
Ca_08057	#NA	PF00271				
**Ca_08069**	**MYB**	PF00249	PF00249			
**Ca_08086**	**WRKY**	PF03106				
**Ca_08103**	**bZIP_1**	PF00170				
Ca_08142	#NA	PF00076	PF00076			
**Ca_08143**	**bZIP_1**	PF00170				
**Ca_08144**	**MYB_related**	PF00249				
Ca_08148	#NA	PF00656				
**Ca_08170**	**MYB**	PF00249	PF00249			
**Ca_08180**	**HB-other**	PF00046				
**Ca_08186**	**Dof**	PF02701				
**Ca_08232**	**ERF**	PF00847				
**Ca_08250**	**GATA**	PF00320				
Ca_08254	#NA	PF00076	PF00076			
**Ca_08255**	**NAC**	PF02365				
**Ca_08257**	**NAC**	PF02365				
Ca_08286	#NA	PF00271				
**Ca_08291**	**ERF**	PF00847				
**Ca_08331**	**ERF**	PF00847				
**Ca_08358**	**MYB_related**	PF00249				
**Ca_08369**	**M-type**	PF00319				
**Ca_08371**	**NAC**	PF02365				
**Ca_08372**	**NAC**	PF02365				
Ca_08387	#NA	PF00072				
Ca_08429	#NA	PF00076				
**Ca_08434**	**MYB_related**	PF00249				
**Ca_08436**	**RAV**	PF00847	PF02362			
**Ca_08440**	**bHLH**	PF00010				
**Ca_08455**	**C2H2**	PF00096				
Ca_08486	#NA	PF01852				
**Ca_08487**	**GATA**	PF00320				
**Ca_08488**	**ARF**	PF02362	PF06507			
**Ca_08506**	**SBP**	PF03110				
Ca_08509	#NA	PF00072				
**Ca_08511**	**CAMTA**	PF03859				
**Ca_08513**	**MIKC**	PF00319	PF01486			
**Ca_08536**	**HSF**	PF00447				
Ca_08542	#NA	PF00076				
Ca_08543	#NA	PF00076				
**Ca_08582**	**ERF**	PF00847				
**Ca_08586**	**bZIP_1**	PF00170				

Accession	TF type	1	2	3	4	5
**Ca_08598**	**MYB_related**	PF00249				
**Ca_08614**	**WRKY**	PF03106				
**Ca_08627**	**HSF**	PF00447				
**Ca_08639**	**MIKC**	PF00319	PF01486			
**Ca_08667**	**HD-ZIP**	PF00046	PF01852			
**Ca_08838**	**HB-PHD**	PF00628	PF00046			
**Ca_08872**	**ARF**	PF02362	PF06507			
Ca_08891	#NA	PF00271				
**Ca_08911**	**ERF**	PF00847				
**Ca_08913**	**ERF**	PF00847				
Ca_08918	#NA	PF00271	PF00271			
**Ca_08924**	**B3**	PF02362				
**Ca_08929**	**bZIP_1**	PF00170				
**Ca_08934**	**M-type**	PF00319				
**Ca_08935**	**M-type**	PF00319				
**Ca_08938**	**M-type**	PF00319				
**Ca_08940**	**M-type**	PF00319				
**Ca_08941**	**M-type**	PF00319				
**Ca_08942**	**M-type**	PF00319				
Ca_08962	#NA	PF00271				
**Ca_08975**	**WRKY**	PF03106	PF03106			
**Ca_08998**	**LBD (AS2/LOB)**	PF03195				
**Ca_09013**	**bZIP_1**	PF00170				
Ca_09032	#NA	PF00628				
Ca_09047	#NA	PF00271				
**Ca_09050**	**ERF**	PF00847				
Ca_09068	#NA	PF03789				
**Ca_09076**	**ERF**	PF00847				
Ca_09080	MYB_related (New)	PF00072	PF00249			
Ca_09081	#NA	PF00628	PF00271			
**Ca_09101**	**WRKY**	PF03106				
**Ca_09110**	**CO-like**	PF00643	PF00643	PF06203		
Ca_09112	#NA	PF00072				
**Ca_09124**	**ERF**	PF00847				
**Ca_09134**	**WRKY**	PF03106				
Ca_09137	#NA	PF00628				
**Ca_09167**	**GATA**	PF00320				
**Ca_09188**	**B3**	PF02362				
**Ca_09200**	**MYB**	PF00249	PF00249			
Ca_09202	#NA	PF03789				
**Ca_09203**	**MYB**	PF00249	PF00249			
**Ca_09212**	**bHLH**	PF00010				
**Ca_09214**	**AP2**	PF00847	PF00847			
**Ca_09216**	**GRF**	PF08880	PF08879			
**Ca_09220**	**bZIP_1**	PF00170				
**Ca_09234**	**Dof**	PF02701				
**Ca_09238**	**Dof**	PF02701				
**Ca_09247**	**LBD (AS2/LOB)**	PF03195				

Accession	TF type	1	2	3	4	5
Ca_09248	LBD (AS2/LOB)	PF03195				
Ca_09273	B3	PF02362				
Ca_09276	ERF	PF00847				
Ca_09277	HSF	PF00447				
Ca_09281	#NA	PF00076	PF00076	PF00076		
Ca_09282	bHLH	PF00010				
Ca_09295	AP2	PF00847	PF00847			
Ca_09302	WRKY	PF03106				
Ca_09341	GRAS	PF03514				
Ca_09342	LBD (AS2/LOB)	PF03195				
Ca_09352	GRAS	PF03514				
Ca_09355	MYB_related	PF00249				
Ca_09365	AP2	PF00847	PF00847			
Ca_09368	MYB	PF00249	PF00249			
Ca_09371	HB-other	PF00046				
Ca_09378	bHLH	PF00010				
Ca_09379	bHLH	PF00010				
Ca_09396	bHLH	PF00010				
Ca_09400	MYB_related	PF00249				
Ca_09407	GRAS	PF03514				
Ca_09421	S1Fa-like	PF04689				
Ca_09442	WRKY	PF03106	PF03106			
Ca_09486	B3	PF02362				
Ca_09495	WRKY	PF03106				
Ca_09530	GRAS	PF03514				
Ca_09538	HB-other	PF00046				
Ca_09547	#NA	PF00076				
Ca_09562	CAMTA	PF03859				
Ca_09578	ERF	PF00847				
Ca_09583	EIL	PF04873				
Ca_09589	#NA	PF00929				
Ca_09600	bZIP_1	PF00170				
Ca_09614	MYB_related	PF00249				
Ca_09628	WRKY	PF03106				
Ca_09638	ERF	PF00847				
Ca_09640	#NA	PF00076	PF00076			
Ca_09655	GRAS	PF03514				
Ca_09667	bHLH	PF00010				
Ca_09673	NAC	PF02365				
Ca_09683	NAC	PF02365				
Ca_09694	WRKY	PF03106	PF03106			
Ca_09696	GRAS	PF03514				
Ca_09784	#NA	PF00072				
Ca_09786	GRAS	PF03514				
Ca_09787	WRKY	PF03106	PF03106			
Ca_09790	#NA	PF00076	PF00076			
Ca_09832	Nin-like	PF02042				
Ca_09871	#NA	PF00076				

Accession	TF type	1	2	3	4	5
**Ca_09877**	**MYB_related**	PF00249				
Ca_09889	#NA	PF01852				
Ca_09953	#NA	PF00271				
Ca_09954	#NA	PF00271				
Ca_09957	#NA	PF00072				
Ca_09981	#NA	PF00076	PF00076			
**Ca_09991**	**GATA**	PF00320				
**Ca_10004**	**GRAS**	PF03514				
**Ca_10012**	**GRF**	PF08880	PF08879			
**Ca_10014**	**MYB**	PF00249	PF00249			
**Ca_10028**	**MYB_related**	PF00249				
**Ca_10039**	**NF-YA**	PF02045				
Ca_10040	#NA	PF00628				
**Ca_10058**	**MYB_related**	PF00249				
Ca_10061	#NA	PF00271				
**Ca_10069**	**MYB**	PF00249	PF00249			
**Ca_10075**	**C2H2**	PF00096				
**Ca_10077**	**LBD (AS2/LOB)**	PF03195				
Ca_10097	#NA	PF00076	PF00076			
**Ca_10106**	**Dof**	PF02701				
**Ca_10121**	**bHLH**	PF00010				
Ca_10153	#NA	PF00271				
Ca_10162	#NA	PF00271				
Ca_10165	#NA	PF00271				
**Ca_10175**	**SRS**	PF05142				
Ca_10190	#NA	PF00076				
Ca_10223	#NA	PF00929				
**Ca_10228**	**bZIP_1**	PF00170				
Ca_10229	#NA	PF00076	PF00076			
**Ca_10236**	**EIL**	PF04873				
**Ca_10247**	**FAR1**	PF03101				
Ca_10289	#NA	PF03789				
Ca_10303	#NA	PF00271				
**Ca_10311**	**GeBP**	PF04504				
**Ca_10316**	**HB-other**	PF00046				
Ca_10347	#NA	PF00076				
**Ca_10352**	**HB-other**	PF00046				
Ca_10376	#NA	PF00271				
Ca_10390	#NA	PF00072				
**Ca_10406**	**HSF**	PF00447				
Ca_10463	#NA	PF00628				
**Ca_10494**	**ERF**	PF00847				
**Ca_10504**	**MYB**	PF00249	PF00249			
**Ca_10510**	**Dof**	PF02701				
**Ca_10514**	**ZF-HD**	PF04770				
**Ca_10515**	**ZF-HD**	PF04770				
**Ca_10517**	**ZF-HD**	PF04770				
**Ca_10518**	**MYB**	PF00249	PF00249			

Accession	TF type	1	2	3	4	5
Ca_10522	#NA	PF00271				
Ca_10527	HB-other	PF00046				
Ca_10533	AP2	PF00847	PF00847			
Ca_10555	MYB	PF00249	PF00249			
Ca_10600	#NA	PF01852				
Ca_10603	GRF	PF08880	PF08879			
Ca_10633	#NA	PF00271				
Ca_10650	BES1	PF05687				
Ca_10730	Nin-like	PF02042				
Ca_10748	ARF	PF02362	PF06507			
Ca_10750	#NA	PF00271				
Ca_10751	#NA	PF00929				
Ca_10755	bZIP_1	PF00170				
Ca_10756	BBR-BPC	PF06217				
Ca_10760	#NA	PF04433				
Ca_10766	#NA	PF00643				
Ca_10767	SRS	PF05142				
Ca_10770	ERF	PF00847				
Ca_10789	B3	PF02362				
Ca_10790	#NA	PF06507				
Ca_10794	ARF	PF02362	PF06507			
Ca_10795	#NA	PF06507				
Ca_10809	MYB_related	PF00249				
Ca_10854	MYB_related	PF00249				
Ca_10855	B3	PF02362	PF02362			
Ca_10856	B3	PF02362				
Ca_10857	B3	PF02362	PF02362	PF02362		
Ca_10886	SRS	PF05142				
Ca_10901	bZIP_1	PF00170				
Ca_10914	CPP	PF03638	PF03638			
Ca_10993	WRKY	PF03106				
Ca_10996	#NA	PF00076				
Ca_11015	bZIP_1	PF00170				
Ca_11021	NAC	PF02365				
Ca_11026	MYB	PF00249	PF00249			
Ca_11060	#NA	PF00076	PF00076	PF00076		
Ca_11064	#NA	PF00076				
Ca_11084	#NA	PF00076	PF00076			
Ca_11128	NAC	PF02365				
Ca_11130	GRAS	PF03514				
Ca_11133	GRAS	PF03514				
Ca_11135	#NA	PF00628				
Ca_11212	GRAS	PF03514				
Ca_11214	E2F/DP	PF02319				
Ca_11215	E2F/DP	PF02319				
Ca_11222	HSF	PF00447				
Ca_11228	MYB	PF00249	PF00249			
Ca_11234	C3H	PF00642				

Accession	TF type	1	2	3	4	5
Ca_11249	#NA	PF00271				
**Ca_11259**	**C3H**	PF00642				
**Ca_11261**	**ERF**	PF00847				
**Ca_11268**	**MYB_related**	PF00249				
**Ca_11272**	**HB-other**	PF00046				
**Ca_11273**	**NAC**	PF02365				
**Ca_11274**	**C2H2**	PF00096				
Ca_11284	#NA	PF00271				
**Ca_11289**	**LBD (AS2/LOB)**	PF03195				
**Ca_11295**	**TCP**	PF03634				
Ca_11309	MYB_related (New)	PF00072	PF00249			
**Ca_11356**	**SBP**	PF03110				
Ca_11358	#NA	PF00072				
**Ca_11361**	**CAMTA**	PF03859				
**Ca_11363**	**MIKC**	PF00319	PF01486			
Ca_11404	#NA	PF00076				
Ca_11418	#NA	PF00271				
Ca_11426	#NA	PF00076				
**Ca_11427**	**MYB_related**	PF00249				
**Ca_11432**	**MYB_related**	PF00249				
Ca_11442	#NA	PF00271				
**Ca_11455**	**bZIP_1**	PF00170				
**Ca_11470**	**HD-ZIP**	PF00046	PF01852			
**Ca_11473**	**MYB_related**	PF00249				
Ca_11483	#NA	PF00076				
**Ca_11487**	**HSF**	PF00447				
**Ca_11494**	**MYB**	PF00249	PF00249			
Ca_11503	#NA	PF00076				
**Ca_11514**	**bHLH**	PF00010				
**Ca_11531**	**bHLH**	PF00010				
Ca_11541	#NA	PF01486				
**Ca_11553**	**Dof**	PF02701				
**Ca_11644**	**WRKY**	PF03106				
Ca_11650	#NA	PF01486				
**Ca_11659**	**M-type**	PF00319				
**Ca_11687**	**NAC**	PF02365				
Ca_11695	#NA	PF00271				
**Ca_11707**	**ERF**	PF00847				
**Ca_11728**	**bHLH**	PF00010				
**Ca_11765**	**ERF**	PF00847				
**Ca_11832**	**MYB**	PF00249	PF00249			
Ca_11842	#NA	PF01852				
Ca_11853	#NA	PF00271				
**Ca_11860**	**CPP**	PF03638	PF03638			
Ca_11886	#NA	PF00271				
**Ca_11891**	**HD-ZIP**	PF00046	PF01852			
**Ca_11909**	**bHLH**	PF00010				
Ca_11928	#NA	PF00271				

Accession	TF type	1	2	3	4	5
Ca_11970	#NA	PF00076				
Ca_11994	#NA	PF00076				
**Ca_12005**	**WRKY**	PF03106	PF03106			
**Ca_12010**	**AP2**	PF00847	PF00847			
Ca_12023	#NA	PF00271				
**Ca_12027**	**S1Fa-like**	PF04689				
**Ca_12028**	**S1Fa-like**	PF04689				
**Ca_12031**	**NAC**	PF02365				
Ca_12066	#NA	PF00076				
Ca_12071	#NA	PF00072				
**Ca_12079**	**ERF**	PF00847				
**Ca_12080**	**ERF**	PF00847				
**Ca_12081**	**ERF**	PF00847				
**Ca_12130**	**MYB**	PF00249	PF00249			
Ca_12170	#NA	PF00628				
**Ca_12172**	**ZF-HD**	PF04770				
**Ca_12183**	**ZF-HD**	PF00447				
**Ca_12194**	**bHLH**	PF00010				
**Ca_12205**	**ERF**	PF00847				
**Ca_12209**	**bHLH**	PF00010				
**Ca_12213**	**LBD (AS2/LOB)**	PF03195				
**Ca_12214**	**LBD (AS2/LOB)**	PF03195				
**Ca_12218**	**ERF**	PF00847				
**Ca_12219**	**ERF**	PF00847				
**Ca_12220**	**ERF**	PF00847				
**Ca_12221**	**ERF**	PF00847				
**Ca_12226**	**MYB**	PF00249	PF00249			
**Ca_12255**	**MYB_related**	PF00249				
Ca_12277	#NA	PF00072				
Ca_12286	#NA	PF00072				
Ca_12296	#NA	PF00072				
**Ca_12298**	**bHLH**	PF00010				
**Ca_12307**	**ZF-HD**	PF04770				
**Ca_12308**	**ZF-HD**	PF04770				
**Ca_12328**	**AP2**	PF00847	PF00847			
**Ca_12334**	**Dof**	PF02701				
**Ca_12352**	**bHLH**	PF00010				
**Ca_12355**	**LBD (AS2/LOB)**	PF03195				
**Ca_12363**	**bHLH**	PF00010				
**Ca_12367**	**C3H**	PF00642	PF00642			
Ca_12374	#NA	PF00076				
**Ca_12377**	**MYB_related**	PF00249				
**Ca_12379**	**YABBY**	PF04690				
**Ca_12381**	**MIKC**	PF00319	PF01486			
**Ca_12391**	**bHLH**	PF00010				
**Ca_12400**	**AP2**	PF00847	PF00847			
**Ca_12416**	**MIKC**	PF00319	PF01486			
**Ca_12420**	**LBD (AS2/LOB)**	PF03195				

Accession	TF type	1	2	3	4	5
**Ca_12446**	**ERF**	PF00847				
Ca_12447	#NA	PF00271				
**Ca_12450**	**LBD (AS2/LOB)**	PF03195				
**Ca_12464**	**LBD (AS2/LOB)**	PF03195				
**Ca_12465**	**ERF**	PF00847				
**Ca_12468**	**MYB**	PF00249	PF00249			
**Ca_12474**	**C3H**	PF00642				
**Ca_12504**	**HB-other**	PF00046				
**Ca_12513**	**MYB_related**	PF00249				
Ca_12537	#NA	PF00628				
Ca_12538	#NA	PF00628				
**Ca_12539**	**HB-other**	PF00046				
**Ca_12575**	**MYB_related**	PF00249				
Ca_12578	#NA	PF00271				
Ca_12597	#NA	PF00076	PF00076			
Ca_12641	#NA	PF04433	PF00249			
Ca_12649	#NA	PF00072				
**Ca_12660**	**NAC**	PF02365				
**Ca_12670**	**bZIP_1**	PF00170				
**Ca_12685**	**EIL**	PF04873				
**Ca_12706**	**C2H2**	PF00096				
**Ca_12720**	**HB-other**	PF00046				
Ca_12721	#NA	PF00271				
Ca_12740	#NA	PF00076				
**Ca_12762**	**MYB**	PF00249	PF00249			
**Ca_12765**	**ERF**	PF00847				
**Ca_12767**	**MYB_related**	PF00249				
**Ca_12769**	**HB-other**	PF00046				
**Ca_12774**	**ERF**	PF00847				
**Ca_12775**	**ERF**	PF00847				
**Ca_12786**	**GATA**	PF00320				
**Ca_12810**	**SRS**	PF05142				
Ca_12825	MYB_related (New)	PF00072	PF00249			
**Ca_12837**	**NAC**	PF02365				
**Ca_12846**	**LSD**	PF06943	PF06943	PF06943		
Ca_12854	#NA	PF00929				
Ca_12859	#NA	PF00628				
**Ca_12866**	**TCP**	PF03634				
Ca_12878	#NA	PF00628				
**Ca_12915**	**WRKY**	PF03106				
**Ca_12923**	**BES1**	PF05687				
**Ca_12925**	**BES1**	PF05687				
Ca_12939	#NA	PF00643				
**Ca_12956**	**CPP**	PF03638	PF03638			
**Ca_12967**	**HSF**	PF00447				
Ca_12996	#NA	PF04433				
**Ca_13012**	**NAC**	PF02365				
Ca_13040	#NA	PF00271				

Accession	TF type	1	2	3	4	5
**Ca_13080**	**bHLH**	PF00010				
**Ca_13084**	**ERF**	PF00847				
Ca_13100	#NA	PF00929				
**Ca_13101**	**GRAS**	PF03514				
**Ca_13102**	**GRAS**	PF03514				
**Ca_13111**	**MIKC**	PF00319	PF01486			
Ca_13113	#NA	PF00076				
**Ca_13133**	**C2H2**	PF00096	PF00096	PF00096	PF00096	
**Ca_13149**	**ZF-HD**	PF04770				
**Ca_13151**	**Nin-like**	PF02042				
**Ca_13157**	**Dof**	PF02701				
**Ca_13160**	**NAC**	PF02365	PF02365			
Ca_13164	#NA	PF00929				
Ca_13165	#NA	PF00072	PF06203			
Ca_13205	#NA	PF00076				
Ca_13214	#NA	PF00076				
**Ca_13216**	**TCP**	PF03634				
**Ca_13221**	**MIKC**	PF00319	PF01486			
**Ca_13222**	**MIKC**	PF00319	PF01486			
**Ca_13228**	**Dof**	PF02701				
**Ca_13231**	**bHLH**	PF00010				
Ca_13232	#NA	PF00628				
**Ca_13236**	**bZIP_1**	PF00170				
Ca_13243	#NA	PF00271				
**Ca_13258**	**NAC**	PF02365				
**Ca_13267**	**MYB_related**	PF00249				
**Ca_13275**	**M-type**	PF00319				
Ca_13280	#NA	PF00076	PF00076			
**Ca_13286**	**MYB**	PF00249	PF00249			
Ca_13289	#NA	PF00076				
**Ca_13295**	**GRAS**	PF03514				
**Ca_13340**	**ERF**	PF00847				
Ca_13379	#NA	PF00271				
Ca_13386	#NA	PF00271				
**Ca_13388**	**LBD (AS2/LOB)**	PF03195				
**Ca_13402**	**bHLH**	PF00010				
**Ca_13435**	**ERF**	PF00847				
Ca_13437	#NA	PF00271				
**Ca_13524**	**WRKY**	PF03106				
Ca_13535	#NA	PF00271				
**Ca_13537**	**NAC**	PF02365				
Ca_13557	#NA	PF00076				
**Ca_13571**	**NAC**	PF02365				
**Ca_13575**	**LBD (AS2/LOB)**	PF03195				
Ca_13576	#NA	PF06203				
Ca_13627	#NA	PF04433	PF00249			
**Ca_13671**	**B3**	PF02362	PF02362			
**Ca_13679**	**Nin-like**	PF02042				

Accession	TF type	1	2	3	4	5
Ca_13686	#NA	PF00076	PF00076			
**Ca_13690**	**CO-like**	PF00643				
**Ca_13713**	**MYB_related**	PF00249				
Ca_13738	#NA	PF08880	PF00271			
Ca_13777	#NA	PF00271				
**Ca_13779**	**MYB**	PF00249	PF00249			
Ca_13792	#NA	PF00076				
Ca_13849	#NA	PF00076	PF00076			
**Ca_13854**	**WRKY**	PF03106	PF03106			
**Ca_13855**	**GRAS**	PF03514				
Ca_13900	#NA	PF00271				
**Ca_13929**	**NAC**	PF02365				
Ca_13974	#NA	PF00929				
**Ca_14003**	**C3H**	PF00642	PF00642			
**Ca_14007**	**bHLH**	PF00010				
Ca_14041	#NA	PF06203				
Ca_14042	#NA	PF00072				
**Ca_14048**	**MYB_related**	PF00249				
Ca_14049	#NA	PF00076				
**Ca_14069**	**C3H**	PF00642				
**Ca_14089**	**ERF**	PF00847				
**Ca_14119**	**MYB**	PF00249	PF00249			
**Ca_14133**	**ERF**	PF00847				
**Ca_14137**	**GRAS**	PF03514				
**Ca_14139**	**bHLH**	PF00010				
**Ca_14144**	**bHLH**	PF00010				
Ca_14148	#NA	PF06203				
**Ca_14170**	**HB-other**	PF00046				
**Ca_14190**	**C3H**	PF00642	PF00642			
Ca_14192	#NA	PF00271				
**Ca_14198**	**MYB_related**	PF00249				
**Ca_14199**	**MYB_related**	PF00249				
**Ca_14201**	**SBP**	PF03110				
**Ca_14204**	**NAC**	PF02365				
**Ca_14212**	**bZIP_1**	PF00170	PF00170			
**Ca_14223**	**bHLH**	PF00010				
**Ca_14231**	**bHLH**	PF00010				
Ca_14236	#NA	PF06203				
**Ca_14247**	**MYB**	PF00249	PF00249			
Ca_14263	#NA	PF00076	PF00642			
Ca_14275	#NA	PF00271				
Ca_14288	#NA	PF00628				
Ca_14295	#NA	PF00076				
**Ca_14329**	**ARF**	PF02362	PF06507			
**Ca_14339**	**MYB_related**	PF00249				
**Ca_14385**	**MYB_related**	PF00249				
**Ca_14390**	**NAC**	PF02365				
**Ca_14410**	**E2F/DP**	PF02319				

Accession	TF type	1	2	3	4	5
**Ca_14440**	**Dof**	PF02701				
Ca_14451	#NA	PF00271				
Ca_14454	#NA	PF00076	PF00076			
**Ca_14455**	**NF-YA**	PF02045				
**Ca_14482**	**bHLH**	PF00010				
Ca_14507	#NA	PF00076				
**Ca_14522**	**MYB**	PF00249	PF00249			
Ca_14547	#NA	PF00271				
**Ca_14560**	**HD-ZIP**	PF00046	PF01852			
Ca_14574	#NA	PF00642	PF00076			
**Ca_14587**	**C2H2**	PF00096				
**Ca_14590**	**ARF**	PF02362	PF06507			
Ca_14593	#NA	PF00628				
**Ca_14605**	**LBD (AS2/LOB)**	PF03195				
**Ca_14610**	**Dof**	PF02701				
Ca_14617	#NA	PF00076				
**Ca_14618**	**AP2**	PF00847	PF00847			
Ca_14621	#NA	PF00072	PF06203			
Ca_14630	#NA	PF06203				
**Ca_14758**	**ERF**	PF00847				
Ca_14780	MYB_related (New)	PF00072	PF00249			
Ca_14796	#NA	PF00271				
**Ca_14797**	**C2H2**	PF00096				
**Ca_14800**	**MYB_related**	PF00249				
**Ca_14804**	**SBP**	PF03110				
**Ca_14807**	**C3H**	PF00642	PF00642			
**Ca_14825**	**B3**	PF02362	PF02362			
Ca_14843	#NA	PF00271				
Ca_14868	#NA	PF08879				
**Ca_14907**	**GATA**	PF00320				
**Ca_14911**	**ERF**	PF00847				
**Ca_14925**	**MYB**	PF00249	PF00249	PF00249		
**Ca_14956**	**M-type**	PF00319				
Ca_14968	GATA (New)	PF06203	PF00320			
**Ca_14981**	**bHLH**	PF00010				
**Ca_15022**	**ERF**	PF00847				
**Ca_15031**	**ERF**	PF00847				
**Ca_15049**	**C3H**	PF00642				
Ca_15050	#NA	PF00076				
**Ca_15055**	**ERF**	PF00847				
**Ca_15064**	**ERF**	PF00847				
Ca_15092	#NA	PF00271				
Ca_15151	MYB_related (New)	PF00072	PF00249			
**Ca_15156**	**MYB_related**	PF00249				
**Ca_15157**	**MYB_related**	PF00249				
**Ca_15158**	**MYB_related**	PF00249				
**Ca_15160**	**NAC**	PF02365				
Ca_15175	#NA	PF00271				

Accession	TF type	1	2	3	4	5
**Ca_15178**	**E2F/DP**	PF02319				
**Ca_15210**	**YABBY**	PF04690				
Ca_15220	#NA	PF00271				
**Ca_15224**	**WRKY**	PF03106				
**Ca_15236**	**NAC**	PF02365				
**Ca_15251**	**WRKY**	PF03106				
**Ca_15301**	**E2F/DP**	PF02319	PF02319			
Ca_15303	#NA	PF00072				
**Ca_15306**	**M-type**	PF00319				
**Ca_15315**	**bHLH**	PF00010				
**Ca_15323**	**LBD (AS2/LOB)**	PF03195				
**Ca_15334**	**HB-other**	PF00046				
**Ca_15343**	**WRKY**	PF03106				
**Ca_15357**	**TCP**	PF03634				
**Ca_15363**	**ZF-HD**	PF04770				
Ca_15369	#NA	PF00076				
Ca_15370	#NA	PF00076				
Ca_15371	#NA	PF00076				
**Ca_15374**	**bHLH**	PF00010				
**Ca_15397**	**bZIP_1**	PF00170				
Ca_15412	#NA	PF00643				
**Ca_15422**	**MYB**	PF00249	PF00249			
**Ca_15423**	**GATA**	PF00320				
**Ca_15515**	**NAC**	PF02365				
**Ca_15518**	**C3H**	PF00642				
**Ca_15538**	**MYB_related**	PF00249				
**Ca_15574**	**NF-YA**	PF02045				
Ca_15587	#NA	PF00271				
Ca_15591	#NA	PF00076	PF00076			
Ca_15594	#NA	PF00076				
**Ca_15603**	**MYB**	PF00249	PF00249			
**Ca_15642**	**LBD (AS2/LOB)**	PF03195				
**Ca_15656**	**M-type**	PF00319				
**Ca_15657**	**bZIP_1**	PF00170				
**Ca_15659**	**B3**	PF02362				
**Ca_15671**	**ERF**	PF00847				
**Ca_15677**	**B3**	PF02362				
**Ca_15694**	**ARF**	PF02362	PF06507			
**Ca_15697**	**EIL**	PF04873				
**Ca_15706**	**bZIP_1**	PF00170				
**Ca_15711**	**AP2**	PF00847	PF00847			
**Ca_15720**	**Dof**	PF02701				
Ca_15731	#NA	PF00271				
Ca_15775	#NA	PF00271				
Ca_15781	#NA	PF00076				
Ca_15788	#NA	PF00628				
**Ca_15792**	**NAC**	PF02365				
**Ca_15839**	**bZIP_1**	PF00170				

Accession	TF type	1	2	3	4	5
Ca_15841	#NA	PF00076				
**Ca_15864**	**MYB_related**	PF00249				
Ca_15874	#NA	PF00076	PF00076			
**Ca_15898**	**GRAS**	PF03514				
Ca_15939	#NA	PF00076	PF00076	PF00076		
**Ca_15961**	**bHLH**	PF00010				
**Ca_15971**	**bZIP_1**	PF00170				
**Ca_16010**	**bHLH**	PF00010				
**Ca_16040**	**bZIP_1**	PF00170				
**Ca_16043**	**GRAS**	PF03514				
**Ca_16044**	**C2H2**	PF00096				
**Ca_16057**	**GATA**	PF00320				
Ca_16097	#NA	PF00076				
**Ca_16122**	**LBD (AS2/LOB)**	PF03195				
**Ca_16131**	**MYB**	PF00249	PF00249			
**Ca_16136**	**bHLH**	PF00010				
Ca_16158	#NA	PF00076	PF00076	PF00076		
**Ca_16164**	**C3H**	PF00642				
**Ca_16180**	**ERF**	PF00847				
**Ca_16227**	**TCP**	PF03634				
Ca_16230	#NA	PF00628				
**Ca_16243**	**SBP**	PF03110				
**Ca_16282**	**MYB_related**	PF00249				
**Ca_16293**	**GRAS**	PF03514				
**Ca_16294**	**GRAS**	PF03514				
**Ca_16355**	**bHLH**	PF00010				
Ca_16365	#NA	PF00076				
**Ca_16371**	**MYB_related**	PF00249				
**Ca_16372**	**Dof**	PF02701				
**Ca_16375**	**bHLH**	PF00010				
**Ca_16376**	**M-type**	PF00319				
**Ca_16379**	**NAC**	PF02365				
**Ca_16386**	**HD-ZIP**	PF00046	PF01852			
**Ca_16393**	**WRKY**	PF03106				
Ca_16396	#NA	PF00076	PF00076			
**Ca_16397**	**ERF**	PF00847				
Ca_16401	MYB_related (New)	PF00072	PF00249			
**Ca_16417**	**TCP**	PF03634				
**Ca_16423**	**M-type**	PF00319				
**Ca_16482**	**C2H2**	PF00096				
**Ca_16486**	**bZIP_1**	PF00170				
Ca_16492	#NA	PF00076	PF00076	PF00076		
**Ca_16514**	**bZIP_1**	PF00170				
**Ca_16539**	**bHLH**	PF00010				
Ca_16569	#NA	PF00628				
**Ca_16578**	**ERF**	PF00847				
**Ca_16593**	**GATA**	PF00320				
**Ca_16631**	**ERF**	PF00847				

Accession	TF type	1	2	3	4	5
**Ca_16645**	**MYB_related**	PF00249				
**Ca_16676**	**GRAS**	PF03514				
**Ca_16677**	**GRAS**	PF03514				
**Ca_16678**	**GRAS**	PF03514				
**Ca_16690**	**Dof**	PF02701				
**Ca_16691**	**HB-other**	PF00046				
Ca_16701	#NA	PF00076				
Ca_16702	#NA	PF00076				
**Ca_16710**	**MYB_related**	PF00249				
**Ca_16744**	**GATA**	PF00320				
**Ca_16749**	**LSD**	PF06943	PF06943	PF06943		
**Ca_16794**	**NF-X1**	PF01422	PF01422			
Ca_16860	#NA	PF00628				
**Ca_16884**	**LBD (AS2/LOB)**	PF03195				
Ca_16898	#NA	PF00271				
Ca_16905	#NA	PF00076				
**Ca_16913**	**MYB**	PF00249	PF00249			
**Ca_16946**	**NAC**	PF02365				
Ca_16954	#NA	PF00271				
**Ca_16982**	**LBD (AS2/LOB)**	PF03195	PF03195			
Ca_17012	#NA	PF00076				
**Ca_17053**	**FAR1**	PF03101				
Ca_17058	#NA	PF06943	PF00656			
**Ca_17079**	**MYB_related**	PF00249				
**Ca_17124**	**C2H2**	PF00096				
**Ca_17136**	**ARF**	PF02362	PF06507			
Ca_17142	#NA	PF00076	PF00076			
**Ca_17152**	**TCP**	PF03634				
Ca_17243	#NA	PF06203				
**Ca_17273**	**WRKY**	PF03106	PF03106			
Ca_17274	#NA	PF00076				
**Ca_17294**	**MYB**	PF00249	PF00249			
**Ca_17354**	**ERF**	PF00847				
Ca_17358	#NA	PF00271				
Ca_17383	#NA	PF01852				
Ca_17397	#NA	PF00271				
Ca_17404	#NA	PF00271				
**Ca_17416**	**bZIP_1**	PF00170				
**Ca_17431**	**MIKC**	PF00319	PF01486			
**Ca_17453**	**bHLH**	PF00010				
**Ca_17470**	**MYB**	PF00249	PF00249			
Ca_17522	GATA (New)	PF06203	PF00320			
Ca_17524	#NA	PF00643				
**Ca_17532**	**AP2**	PF00847	PF00847			
**Ca_17562**	**C2H2**	PF00096				
Ca_17565	#NA	PF00076				
**Ca_17591**	**bHLH**	PF00010				
**Ca_17597**	**MYB_related**	PF00249				

Accession	TF type	1	2	3	4	5
Ca_17598	MYB_related	PF00249				
Ca_17603	bHLH	PF00010				
Ca_17608	HB-other	PF00046				
Ca_17620	#NA	PF00076				
Ca_17624	ARF	PF02362	PF06507			
Ca_17627	MYB	PF00249	PF00249			
Ca_17636	ARF	PF02362	PF06507			
Ca_17638	ERF	PF00847				
Ca_17639	bHLH	PF00010				
Ca_17657	MYB	PF00249	PF00249			
Ca_17701	#NA	PF00271				
Ca_17707	#NA	PF00628				
Ca_17759	WRKY	PF03106				
Ca_17787	bZIP_1	PF00170				
Ca_17823	C3H	PF00642	PF00642	PF00642	PF00642	PF00642
Ca_17824	ERF	PF00847				
Ca_17883	ERF	PF00847				
Ca_17916	MYB_related	PF00249				
Ca_17934	C3H	PF00642				
Ca_17967	#NA	PF00929				
Ca_17971	bZIP_1	PF00170				
Ca_17984	SRS	PF05142				
Ca_17994	#NA	PF00072				
Ca_18062	#NA	PF00628				
Ca_18077	MYB_related	PF00249				
Ca_18079	Dof	PF02701				
Ca_18082	M-type	PF00319				
Ca_18090	NAC	PF02365				
Ca_18110	bHLH	PF00010				
Ca_18118	ZF-HD	PF04770				
Ca_18127	ERF	PF00847				
Ca_18152	#NA	PF03789				
Ca_18169	#NA	PF01852				
Ca_18171	NAC	PF02365				
Ca_18173	#NA	PF00628				
Ca_18177	ZF-HD	PF04770				
Ca_18206	LBD (AS2/LOB)	PF03195				
Ca_18243	#NA	PF00072	PF06203			
Ca_18285	bHLH	PF00010				
Ca_18366	bHLH	PF00010				
Ca_18387	ERF	PF00847				
Ca_18416	#NA	PF00628				
Ca_18530	M-type	PF00319				
Ca_18533	YABBY	PF04690				
Ca_18551	#NA	PF00076				
Ca_18591	SBP	PF03110				
Ca_18605	LSD	PF06943	PF06943	PF06943		
Ca_18623	HB-other	PF00046				

Accession	TF type	1	2	3	4	5
Ca_18628	#NA	PF00076	PF00076			
Ca_18672	#NA	PF00271	PF00271			
**Ca_18688**	**bHLH**	PF00010				
**Ca_18699**	**MYB**	PF00249	PF00249			
**Ca_18705**	**ZF-HD**	PF04770				
Ca_18725	#NA	PF00076				
Ca_18733	#NA	PF01486				
**Ca_18738**	**B3**	PF02362				
**Ca_18739**	**B3**	PF02362	PF02362			
**Ca_18745**	**bZIP_1**	PF00170				
**Ca_18749**	**NAC**	PF02365				
**Ca_18764**	**TCP**	PF03634				
**Ca_18774**	**bHLH**	PF00010				
**Ca_18777**	**HB-other**	PF00046				
**Ca_18788**	**Whirly**	PF08536				
Ca_18802	#NA	PF00076				
**Ca_18845**	**M-type**	PF00319				
Ca_18871	#NA	PF00076	PF00076			
**Ca_18894**	**AP2**	PF00847	PF00847			
**Ca_18899**	**bHLH**	PF00010				
**Ca_18932**	**WRKY**	PF03106				
**Ca_18942**	**SBP**	PF03110				
**Ca_18948**	**ERF**	PF00847				
**Ca_18954**	**MYB**	PF00249	PF00249			
**Ca_19027**	**ERF**	PF00847				
**Ca_19041**	**MYB**	PF00249	PF00249			
**Ca_19135**	**ERF**	PF00847				
Ca_19140	#NA	PF00271				
**Ca_19144**	**NAC**	PF02365				
**Ca_19147**	**MYB_related**	PF00249				
**Ca_19232**	**MYB_related**	PF00249				
Ca_19242	#NA	PF00076	PF00076	PF00076		
**Ca_19269**	**bHLH**	PF00010				
Ca_19271	#NA	PF00076	PF00076	PF00076	PF00076	
**Ca_19272**	**HB-other**	PF00046				
Ca_19288	#NA	PF00271				
**Ca_19289**	**ARF**	PF02362	PF06507			
**Ca_19295**	**ERF**	PF00847				
**Ca_19296**	**ERF**	PF00847				
**Ca_19297**	**ERF**	PF00847				
**Ca_19327**	**MYB_related**	PF00249				
Ca_19356	#NA	PF00271				
**Ca_19383**	**HB-other**	PF00046				
**Ca_19386**	**HB-other**	PF00046				
**Ca_19393**	**MYB**	PF00249	PF00249			
Ca_19405	#NA	PF00271				
**Ca_19415**	**MYB_related**	PF00249				
**Ca_19433**	**Dof**	PF02701				

Accession	TF type	1	2	3	4	5
Ca_19440	#NA	PF00271				
Ca_19441	#NA	PF01486				
**Ca_19449**	**SBP**	PF03110				
**Ca_19481**	**bHLH**	PF00010				
**Ca_19492**	**HB-other**	PF00046				
**Ca_19501**	**WRKY**	PF03106				
**Ca_19529**	**bHLH**	PF00010				
Ca_19588	#NA	PF08879	PF08879			
**Ca_19597**	**LBD (AS2/LOB)**	PF03195				
**Ca_19598**	**LBD (AS2/LOB)**	PF03195				
**Ca_19630**	**MYB**	PF00249	PF00249			
**Ca_19648**	**Dof**	PF02701				
**Ca_19654**	**NAC**	PF02365				
**Ca_19689**	**bZIP_1**	PF00170				
**Ca_19697**	**HD-ZIP**	PF00046	PF01852			
**Ca_19700**	**MYB**	PF00249	PF00249			
**Ca_19701**	**HB-other**	PF00046				
**Ca_19705**	**Dof**	PF02701				
**Ca_19716**	**LBD (AS2/LOB)**	PF03195				
Ca_19811	#NA	PF00076				
**Ca_19863**	**bHLH**	PF00010				
**Ca_19916**	**ZF-HD**	PF04770				
**Ca_20021**	**TCP**	PF03634				
**Ca_20022**	**TCP**	PF03634				
**Ca_20081**	**MYB**	PF00249	PF00249			
**Ca_20090**	**GeBP**	PF04504				
**Ca_20117**	**GRAS**	PF03514				
**Ca_20127**	**ERF**	PF00847				
**Ca_20151**	**HB-other**	PF00046				
**Ca_20200**	**LBD (AS2/LOB)**	PF03195				
**Ca_20223**	**GRAS**	PF03514				
Ca_20246	#NA	PF00271				
**Ca_20250**	**NF-YA**	PF02045				
Ca_20256	#NA	PF00076	PF00076	PF00076		
Ca_20257	#NA	PF00271				
**Ca_20349**	**ERF**	PF00847				
Ca_20367	#NA	PF00076				
**Ca_20380**	**MYB**	PF00249	PF00249			
Ca_20387	#NA	PF00072				
Ca_20395	#NA	PF00271				
**Ca_20428**	**LBD (AS2/LOB)**	PF03195				
**Ca_20429**	**LBD (AS2/LOB)**	PF03195				
**Ca_20437**	**MYB**	PF00249	PF00249			
Ca_20443	#NA	PF00076	PF00076			
Ca_20446	#NA	PF00076				
Ca_20447	#NA	PF00076				
Ca_20448	#NA	PF00076				
Ca_20458	#NA	PF06203				

Accession	TF type	1	2	3	4	5
Ca_20482	#NA	PF00271				
Ca_20487	#NA	PF00271				
**Ca_20508**	**WRKY**	PF03106	PF03106			
**Ca_20616**	**bHLH**	PF00010				
**Ca_20636**	**HB-other**	PF00046				
Ca_20647	#NA	PF01486				
**Ca_20649**	**MIKC**	PF00319	PF01486			
**Ca_20675**	**WRKY**	PF03106				
**Ca_20689**	**AP2**	PF00847	PF00847			
Ca_20739	#NA	PF00076				
Ca_20783	#NA	PF00271				
Ca_20820	#NA	PF00076	PF00076	PF00076		
Ca_20965	#NA	PF00076				
**Ca_20968**	**ERF**	PF00847				
**Ca_20988**	**NAC**	PF02365				
**Ca_21021**	**Dof**	PF02701				
Ca_21100	#NA	PF00076				
**Ca_21127**	**MYB**	PF00249	PF00249			
**Ca_21136**	**MYB_related**	PF00249				
**Ca_21138**	**GRAS**	PF03514				
**Ca_21346**	**ZF-HD**	PF04770				
**Ca_21348**	**ZF-HD**	PF04770				
**Ca_21349**	**ZF-HD**	PF04770				
**Ca_21404**	**HB-other**	PF00046				
**Ca_21411**	**MYB_related**	PF00249				
**Ca_21472**	**YABBY**	PF04690				
**Ca_21576**	**bHLH**	PF00010				
**Ca_21669**	**WRKY**	PF03106				
Ca_21939	MYB_related (New)	PF00072	PF00249			
**Ca_21948**	**ARF**	PF02362	PF06507			
**Ca_22008**	**bHLH**	PF00010				
Ca_22019	#NA	PF08879				
**Ca_22021**	**MYB**	PF00249	PF00249			
**Ca_22043**	**bHLH**	PF00010				
Ca_22065	#NA	PF00628				
**Ca_22078**	**MYB**	PF00249	PF00249			
**Ca_22090**	**WRKY**	PF03106	PF03106			
**Ca_22099**	**WRKY**	PF03106				
**Ca_22148**	**CO-like**	PF00643	PF06203			
Ca_22223	#NA	PF00271				
**Ca_22233**	**bHLH**	PF00010				
Ca_22248	#NA	PF00076				
**Ca_22309**	**bHLH**	PF00010				
Ca_22316	#NA	PF00076				
Ca_22358	#NA	PF00271				
**Ca_22367**	**MYB**	PF00249	PF00249			
**Ca_22370**	**NAC**	PF02365				
**Ca_22383**	**Dof**	PF02701				

Accession	TF type	1	2	3	4	5
**Ca_22438**	**MIKC**	PF00319	PF01486			
Ca_22462	#NA	PF00271				
**Ca_22502**	**ERF**	PF00847				
Ca_22513	#NA	PF00271				
**Ca_22526**	**SBP**	PF03110				
**Ca_22532**	**NAC**	PF02365				
**Ca_22534**	**ERF**	PF00847				
Ca_22554	#NA	PF00929				
Ca_22567	#NA	PF00076	PF00076			
Ca_22571	#NA	PF06203				
Ca_22631	#NA	PF06203				
**Ca_22732**	**CO-like**	PF00643	PF06203			
**Ca_22733**	**ERF**	PF00847				
**Ca_22771**	**GRAS**	PF03514	PF03514			
**Ca_22917**	**WRKY**	PF03106				
**Ca_22941**	**NAC**	PF02365				
**Ca_23042**	**SRS**	PF05142				
**Ca_23054**	**ZF-HD**	PF04770				
**Ca_23149**	**GRF**	PF08880	PF08879			
**Ca_23163**	**MYB_related**	PF00249				
**Ca_23170**	**ERF**	PF00847				
Ca_23176	#NA	PF00271				
**Ca_23230**	**NAC**	PF02365				
**Ca_23246**	**Dof**	PF02701				
**Ca_23296**	**ARF**	PF02362	PF06507			
**Ca_23324**	**HD-ZIP**	PF00046	PF01852	PF01852		
**Ca_23325**	**GATA**	PF00320				
**Ca_23356**	**C3H**	PF00642				
Ca_23422	#NA	PF00072				
**Ca_23494**	**GRAS**	PF03514				
Ca_23520	#NA	PF00271				
**Ca_23533**	**MYB_related**	PF00249				
**Ca_23537**	**NAC**	PF02365				
Ca_23539	#NA	PF00628				
Ca_23540	#NA	PF00656				
Ca_23608	#NA	PF00656				
Ca_23609	#NA	PF00628				
Ca_23643	#NA	PF01852				
Ca_23684	#NA	PF00076				
**Ca_23693**	**bHLH**	PF00010				
**Ca_23694**	**bHLH**	PF00010				
**Ca_23745**	**EIL**	PF04873				
**Ca_23748**	**bZIP_1**	PF00170				
**Ca_23892**	**bHLH**	PF00010				
Ca_23902	#NA	PF00271				
Ca_23911	#NA	PF00628				
Ca_23964	#NA	PF00271				
**Ca_24009**	**MYB_related**	PF00249				

Accession	TF type	1	2	3	4	5
Ca_24030	#NA	PF00271				
Ca_24049	#NA	PF00628				
Ca_24261	#NA	PF00076				
**Ca_24270**	**bHLH**	PF00010				
**Ca_24372**	**LBD (AS2/LOB)**	PF03195				
**Ca_24417**	**GRAS**	PF03514				
**Ca_24539**	**MIKC**	PF00319	PF01486			
**Ca_24550**	**MYB**	PF00249	PF00249			
**Ca_24649**	**MIKC**	PF00319	PF01486			
Ca_24650	#NA	PF01486				
**Ca_24651**	**M-type**	PF00319				
**Ca_24655**	**SBP**	PF03110				
Ca_24757	#NA	PF00076				
**Ca_24772**	**NAC**	PF02365				
**Ca_24775**	**bHLH**	PF00010				
**Ca_24948**	**TCP**	PF03634				
Ca_25061	#NA	PF04433				
**Ca_25078**	**WRKY**	PF03106				
**Ca_25086**	**M-type**	PF00319				
**Ca_25098**	**C3H**	PF00642				
Ca_25545	#NA	PF00271				
Ca_25713	#NA	PF01852				
Ca_25717	#NA	PF00628				
Ca_25835	#NA	PF00271				
**Ca_25882**	**HB-other**	PF00046				
**Ca_25981**	**YABBY**	PF04690				
**Ca_26120**	**GATA**	PF00320				
**Ca_26121**	**ARF**	PF02362	PF06507			
**Ca_26279**	**GRAS**	PF03514				
**Ca_26354**	**M-type**	PF00319				
Ca_26505	#NA	PF00271				
**Ca_26862**	**C3H**	PF00642				
Ca_26865	#NA	PF00076				
Ca_27452	#NA	PF06203				
**Ca_27693**	**bZIP_1**	PF00170				
Ca_27703	#NA	PF00072				
**Ca_00096**	**Tubby***	PF00646	PF01167			
**Ca_00536**	**Tubby***	PF00646	PF01167			
**Ca_02030**	**Tubby***	PF00646	PF01167			
Ca_02885	#NA	PF04525				
Ca_02936	#NA	PF01167				
**Ca_03429**	**Tubby***	PF00646	PF01167			
**Ca_04384**	**Tubby***	PF00646	PF01167			
Ca_05729	#NA	PF04525				
Ca_05787	#NA	PF04525				
Ca_06022	#NA	PF00069	PF00560	PF00560	PF00560	PF04525
Ca_09090	#NA	PF04525				
**Ca_10800**	**Tubby***	PF00646	PF01167			

Accession	TF type	1	2	3	4	5
**Ca_12896**	**Tubby***	PF00646	PF01167			
**Ca_15312**	**Tubby***	PF00646	PF01167			
**Ca_16064**	**Tubby***	PF00646	PF01167			
Ca_16185	#NA	PF04525				
Ca_17276	#NA	PF04525				
Ca_19189	#NA	PF04525				
Ca_22350	#NA	PF04525				

Color coding

**Ca_00016**	Included
Ca_00010	Discarded
Ca_01471	New Pattern

DNA binding domain

PF00847	AP2
PF02362	B3
PF06217	GAGA_bind
PF05687	DUF822
PF00010	HLH
PF00170	bZIP_1
PF00643	zf-B_box
PF02701	zf-Dof
PF00320	GATA
PF06943	zf-LSD1
PF04690	YABBY
PF00096	zf-C2H2
PF00642	zf-CCCH
PF03859	CG-1
PF03638	TCR
PF02319	E2F_TDP
PF04873	EIN3
PF03101	FAR1
PF04504	DUF573
PF03514	GRAS
PF08879	WRC
PF00046	Homeobox
PF00447	HSF_DNA-bind
PF03195	DUF260
PF01698	FLO_LFY
PF00319	SRF-TF
PF00249	Myb_DNA-binding
PF02365	NAM
PF01422	zf-NF-X1
PF02045	CBFB_NFYA
PF02042	RWP-RK
PF08744	NOZZLE
PF04689	S1FA
PF03110	SBP
PF05142	DUF702
PF03634	TCP
PF08536	Whirly
PF03106	WRKY
PF04770	ZF-HD_dimer
PF00643	zf-B_box
PF01167	Tub
PF04525	Tubby C 2

Aux domain

PF06507	Auxin_resp
PF06203	CCT
PF03789	ELK
PF00646	F-Box
PF01486	K-box
PF00628	PHD
PF08880	QLQ
PF00072	Response_reg
PF01852	START

Forbidden domain

PF00271	Helicase_C
PF00656	Peptidase_C14
PF00929	RNase_T
PF00076	RRM_1
PF04433	SWIRM
