# Supplementary material for: CicerTransDB 1.0: a resource for expression and functional study of chickpea transcription factors
Source: BMC Plant Biol. 2016 Jul 29;16:169. doi: 10.1186/s12870-016-0860-y (PMC4966752; doi:10.1186/s12870-016-0860-y)
Supplement: Additional file 2: Figure S1. — Diagram showing CicerTransDB webserver workflow. Bold red lines show user accessible pipeline. Blue and green dotted lines show workflow running through website PHP without user’s interaction. Blue lines are normal PHP data, while green lines are secured ones. (PDF 39 kb) [file 12870_2016_860_MOESM2_ESM.pdf]

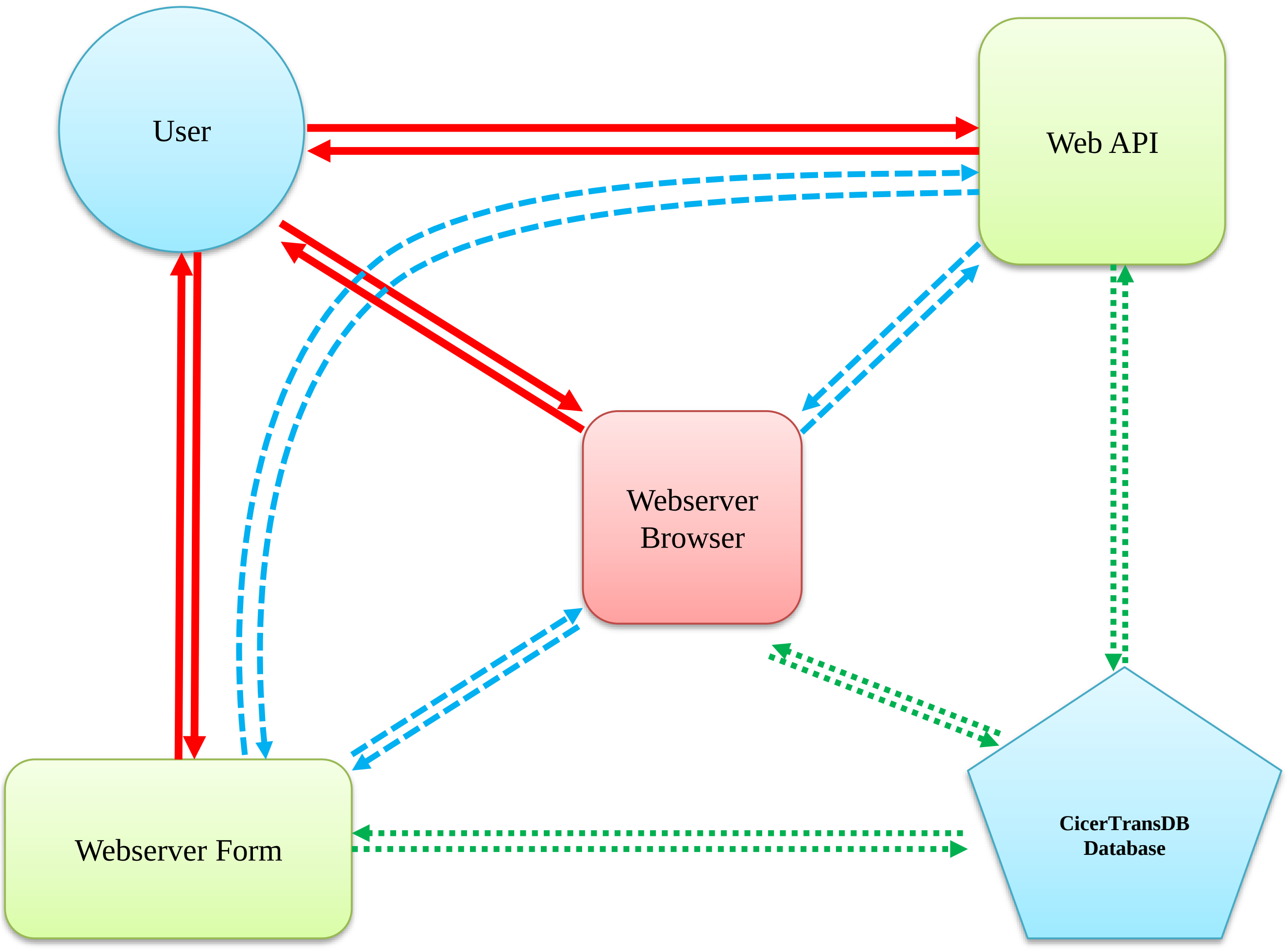

Additional file 2: Figure S1. Diagram showing CicerTransDB webserver workflow. Bold red lines show user accessible pipeline. Blue and green dotted lines show workflow running through website PHP without user's interaction. Blue lines are normal PHP data while green lines are secured ones.
