# Supplementary material for: CicerTransDB 1.0: a resource for expression and functional study of chickpea transcription factors
Source: BMC Plant Biol. 2016 Jul 29;16:169. doi: 10.1186/s12870-016-0860-y (PMC4966752; doi:10.1186/s12870-016-0860-y)
Supplement: Additional file 3: Figure S2. — A bar chart showing distribution of transcription factors in chickpea genome on individual chromosomes and line chart showing average density of transcription factors in each chromosome. The data shows count of all TF types. Individual family distribution graph has been included in the database website. (PDF 26 kb) [file 12870_2016_860_MOESM3_ESM.pdf]

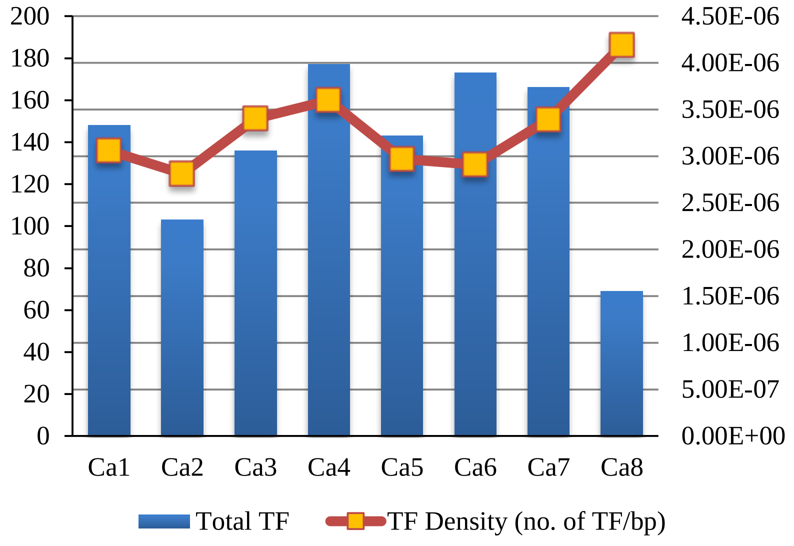

Additional file 3: Figure S2. A bar chart showing distribution of transcription factors in chickpea genome on individual chromosomes and line chart showing average density of transcription factors in each chromosome. The data shows count of all TF types. Individual family distribution graph has been included in the database website.
