# Supplementary material for: CicerTransDB 1.0: a resource for expression and functional study of chickpea transcription factors
Source: BMC Plant Biol. 2016 Jul 29;16:169. doi: 10.1186/s12870-016-0860-y (PMC4966752; doi:10.1186/s12870-016-0860-y)
Supplement: Additional file 4: Table S2. — Distribution of TF family genes across the chromosomes. (PDF 42 kb) [file 12870_2016_860_MOESM4_ESM.pdf]

Additional file 4: Table S2. Distribution of TF family genes across the chromosomes.

	Ca1	Ca2	Ca3	Ca4	Ca5	Ca6	Ca7	Ca8	Total
**AP2**	2	2	2	1	1	3	7	2	**20**
**ARF**	5	2	1	4	2	7	3	0	**24**
**B3**	1	2	4	6	5	3	1	0	**22**
**BBR-BPC**	0	0	0	1	0	1	0	0	**2**
**BES1**	2	0	1	0	1	0	0	2	**6**
**bHLH**	12	10	14	20	12	16	18	8	**110**
**bZIP_1**	3	4	10	10	9	7	6	1	**50**
**C2H2**	2	1	2	7	3	4	2	0	**21**
**C3H**	6	2	5	6	5	5	5	2	**36**
**CAMTA**	1	0	0	0	1	3	1	0	**6**
**CO-like**	0	0	2	2	2	1	1	1	**9**
**CPP**	0	0	0	1	1	1	0	2	**5**
**Dof**	7	3	4	2	2	6	7	1	**32**
**E2F-DP**	0	0	0	1	1	3	1	0	**6**
**EIL**	1	1	0	0	3	1	1	0	**7**
**ERF**	15	7	16	24	12	9	13	10	**106**
**FAR1**	4	1	1	0	0	0	0	0	**6**
**GATA**	2	1	2	4	4	5	6	3	**27**
**GeBP**	1	0	1	2	0	1	0	1	**6**
**GRAS**	8	2	4	5	2	13	6	2	**42**
**GRF**	0	0	1	1	1	1	1	1	**6**
**HB-other**	4	6	5	4	6	9	6	4	**44**
**HB-PHD**	1	0	0	1	0	0	0	0	**2**
**HD-ZIP**	8	1	1	2	1	2	2	2	**19**
**HSF**	1	0	1	3	2	4	2	4	**17**
**LBD (AS2-LOB)**	3	8	5	3	5	4	9	3	**40**
**LFY**	0	0	0	0	1	0	0	0	**1**
**LSD**	1	0	0	0	0	2	1	0	**4**
**M-type**	1	6	1	8	9	5	3	0	**33**
**MIKC**	1	2	2	3	3	3	4	1	**19**
**MYB**	7	8	13	14	7	9	13	3	**74**
**MYB_related**	13	8	11	15	16	14	14	7	**98**
**NAC**	12	7	7	7	8	14	3	5	**63**
**NF-X1**	0	0	0	0	0	0	0	1	**1**
**NF-YA**	2	0	1	1	1	0	2	0	**7**
**Nin-like**	1	1	0	1	3	1	1	1	**9**
**NZZ-SPL**	1	0	0	0	0	0	0	0	**1**
**RAV**	0	0	0	1	0	0	0	0	**1**
**S1Fa-like**	0	0	3	1	0	0	0	0	**4**
**SBP**	5	1	2	3	2	2	2	0	**17**
**SRS**	0	1	0	2	0	0	3	1	**7**
**TCP**	4	1	0	3	1	2	5	0	**16**
**Tubby**	3	0	0	3	0	0	2	1	**9**
**Whirly**	0	1	0	0	0	0	0	0	**1**
**WRKY**	10	7	10	7	8	10	10	0	**62**
**YABBY**	1	2	0	0	0	2	2	0	**7**
**ZF-HD**	0	5	4	1	3	0	5	1	**19**
**Total TF**	**151**	**103**	**136**	**180**	**143**	**173**	**168**	**70**	
